# Upcycling of Waste Poly(ethylene Terephthalate): Ammonolysis Kinetics of Model Bis(2‐Hydroxyethyl Terephthalate) and Particle Size Effects in Polymeric Substrates

**DOI:** 10.1002/cssc.202500509

**Published:** 2025-06-30

**Authors:** Richard‐Joseph L. Peterson, Elanna P. Neppel, Daniel Holmes, P. Anh Trinh, Robert Y. Ofoli, John R. Dorgan

**Affiliations:** ^1^ Chemical Engineering and Materials Science Department Michigan State University East Lansing MI 48823 USA; ^2^ Chemistry Department Michigan State University East Lansing MI 48823 USA; ^3^ Present address: School of Chemical and Biomolecular Engineering Georgia Institute of Technology Atlanta GA 30332 USA

**Keywords:** ammonolysis, diffusion, plastic waste, polymers, reaction kinetics, recycling, upcycling

## Abstract

Chemical upcycling of poly(ethylene terephthalate) (PET) through ammonolysis is pursued. Model studies are conducted on bis(2‐hydroxyethyl) terephthalate reacting with ammonia in the presence of excess ethylene glycol (EG) to form terephthalamide. Reactions conducted over the range of temperatures from 50 to 125 °C yield pre‐exponential factors of *A*_1_ = 758 ± 120 L mol^−1^ h^−^^1^/*A*_2_ = 1033 ± 220 L mol^−1^ h^−^^1^ and activation energies of *E*_*a*1_ = 22.1 ± 1.5 kJ mol^−1^ and *Ea*_2_ = 26.5 ± 2.1 kJ mol^−1^. Diffusional limitations are explored using particle sizes in the ranges of 1800–2500 μm, 250–600 μm, and 150–250 μm. Data from the particle size experiments is combined with the rate constants determined from the model studies to construct an effectiveness factor. Diffusivity is used as a fitting parameter, resulting in an estimated value of 1.37 ± 0.48 E‐7 cm^2^ s^−1^ for ammonia in PET at 100 °C, which compares well to related literature values. Diffusional limitations are of profound importance; for particles of only 1 millimeter in average thickness, the conversion rate is decreased by a factor of ten! The comprehensive understanding of the ammonolysis of PET in EG provided can play an important role in improving material reuse and fostering a more circular economy.

## Graphical abstract

Chemical upcycling of poly(ethylene terephthalate) (PET) through ammonolysis is pursued. Model studies convert bis(2‐hydroxyethyl) terephthalate to terephthalamide. Diffusional limitations are explored using varying PET particle sizes and are profoundly important; for particles only 1 millimeter thick, the conversion rate decreases by a factor of ten! The comprehensive understanding of PET ammonolysis should help foster a more circular economy.

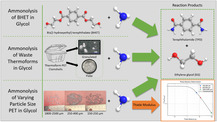

## 1 Introduction

In response to the limited success of traditional recycling methods, innovative chemical recycling and upcycling technologies are of increasing interest. In chemical recycling, plastics are broken down into monomers that can be repolymerized into high‐quality virgin materials. For example, poly(ethylene terephthalate) (PET) is commercially recycled using glycolysis and methanolysis.^[^ ^1^, ^2^, ^3^, ^4^, ^5^, ^6^, ^7^, ^8^, ^9^, ^10^, ^11^ ^]^ Work continues to make simple hydrolysis economically viable.^[^ ^12^, ^13^ ^]^ Less economic approaches like enzyme catalysis also continue to be investigated.^[^ ^14^, ^15^ ^]^ In chemical upcycling, the resulting products are more valuable than the pristine monomers. A relevant example of upcycling is found in the production of diamines by aminolysis of PET.^[^ ^16^, ^17^, ^18^, ^19^, ^20^, ^21^ ^]^ As shown by these examples, PET is a good candidate for chemical recycling because it contains a valuable aromatic group and its ester bonds can be cleaved by many types of reactants.^[^ ^22^ ^]^

Ammonolysis represents a potential chemical upcycling process for PET with considerable benefits. In this approach, PET is reacted with ammonia to generate terephthalamide (TPD).^[^ ^23^, ^24^, ^25^ ^]^ TPD can then be converted to p‐phenylenediamine and other value‐added monomers. Previous work by Zengel and Bergfeld investigated ammonolysis of PET in the presence of excess ethylene glycol (EG).^[^ ^26^, ^27^, ^28^ ^]^ Their industrially oriented process utilizes ammonia gas fed to a stirred reactor containing a slurry of PET pellets dispersed in EG. Reaction kinetics are described only in terms of conversion to TPD as a function of time; no detailed chemical kinetics data are presented. Importantly, this previous work does not address how the conversion rate is impacted by different particle sizes. In contrast, this study establishes fundamental reaction rates using bis(2‐hydroxyethyl) terephthalate (BHET) as a model compound. The premise of using BHET as the model compound for ammonolysis of PET is that the ester linkages between the aromatic ring and the ethylene units have a similar electronic structure to the ester linkages in the PET backbone. Similarly, when one of the two ester bonds is converted to an amide, the intermediate, 2‐hydroxyethyl 4‐carbamoylbenzoate (HCB), serves as a model for one of the PET chain ends. Determination of the reaction rate constants for the model BHET enables investigation of particle size effects on the ammonolysis of PET. Conversion data on different PET particle sizes is analyzed in terms of the Thiele modulus and an effectiveness factor. The outcome of the study is all the fundamental information required to perform a detailed, large‐scale reactor design for the ammonolysis of any form (thermoforms, bottles, fibers, off‐spec pellets) of PET to TPD. The detailed design is the subject of a continuation of the present investigation.

## 2 Materials and Methods

### 2.1 Dissolution of Ammonia in EG

**Figure 1:**
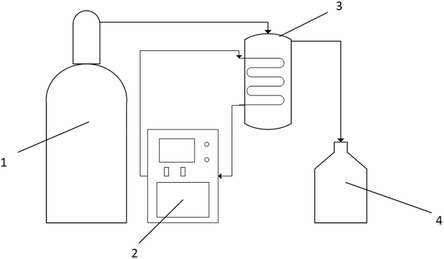
A system used to dissolve ammonia in ethylene glycol. The apparatus consists of 1) a regulated ammonia tank, 2) a temperature controller, 3) chilled ethylene glycol, and 4) a container filled with ethylene glycol to trap excess ammonia.

Ammonia gas is toxic and corrosive. To mitigate exposure risks, EG was presaturated with ammonia. To avoid skin and eye contact, appropriate personal protective equipment (chemically resistant gloves and goggles) was always worn. This saturation procedure was performed in a chemical fume hood. The method for dissolving ammonia in EG was based on the procedure outlined by Zhou et al. and illustrated in **Figure** **1**.^[^ ^29^ ^]^ Ammonia was bubbled into a chilled vessel containing EG until saturation was achieved. Excess ammonia gas was trapped in a secondary container of EG.

### 2.2 Ammonolysis of BHET

Experiments were conducted using a Parr 5000 multi‐reactor system, monitored and controlled by a Parr 4870 process controller. This system comprises six individual T316 stainless steel reactors, each capable of being set to different temperatures and pressures. Separate reactors were quenched and harvested at varying times. Details appear in the Supporting Information (SI).

The BHET reaction procedure was as follows. First, BHET was crushed into a fine powder using a mortar and pestle. Secondly, one gram (1.0 g) of the powdered BHET was placed in a glass liner of a 75 mL Parr reactor along with a magnetic stir bar. Next, 10 mL of the presaturated ammonia in EG solution was added. Reaction conditions are listed in **Table** **1**. Ammonia concentrations were set by the temperature‐dependent saturation limit of pre‐dissolved ammonia in liquid EG. The upper temperature of 125 °C was selected to avoid the side reaction of EG with ammonia, which is known to begin around 150 °C. After loading, the reactors were connected to a gas manifold and pressurized with 40 bar of ultra‐high purity (99.999%) nitrogen (Air‐Gas). The stir rate was set to 800 RPM, and the Parr temperature controller unit was used to bring each reactor up to the setpoint temperature of 50 °C, 75 °C, 100 °C, or 125 °C. Product mixtures were harvested at intervals of 0.8 h, 1.5 h, 2.3 h, 3.0 h, 3.8 h, 4.5 h, 6.0 h, 9.0 h, and 24.0 h.

**Table 1:** Reaction conditions.

Reaction setpoint temperature	Ammonia molarity	Ammonia mole fraction
50 °C	10.9 m	0.45
75 °C	5.5 m	0.23
100 °C	2.7 m	0.11
125 °C	2.7 m	0.11

Reactions were halted by thermal quenching followed by the addition of water. At the end of each reaction, the entire reactor was quenched in an ice bath until its internal thermocouple read 5 °C. The pressure was vented. Solids were then separated from liquids via vacuum filtration. Aromatic products remained dissolved in the EG solutions; liquid NMR samples were prepared in an NMR tube by mixing 0.3 mL of the EG solution with 0.3 mL of DMSO‐d6. Solid products were washed with excess water and dried at 60 °C overnight. Both solid and liquid samples were analyzed using a 500 MHz proton NMR in deuterated dimethyl sulfoxide (DMSO‐d6), employing a two‐second relaxation delay and 32 scans.

Mole fractions were calculated using NMR. Integrations from the proton NMR, combined with material balances on the aromatic species, were used to determine the mole fractions for each sample. A detailed example calculation is provided in the SI.

### 2.3 Ammonolysis of Post‐Consumer PET Waste

Post‐consumer thermoform PET waste was converted to TPD at 100 °C. The thermoform waste consisted of clamshell containers obtained at the MSU campus recycling center. The thermoform PET was cut into ≈1 cm^2^ squares, referred to as “flakes.” Some flakes were set aside for conversion to TPD, while others were further reduced in size to 150–250 μm using a commercial blender (Waring model CB15). PET (1.3 g) was placed in glass liners of the 75 mL Parr reactors, and the procedure used for BHET was repeated. The number average molecular weight as a function of time was monitored by end group analysis using proton NMR in trifluoroacetic acid. Residual solids were dissolved in 10 mL of DMSO to separate out the monomers (BHET, HCB, and TPD) from the remaining PET. After filtration, 30 mL of water was added to the DMSO solution containing the desired monomers. This water addition led to recrystallization, and the resulting solids were collected by filtration.

### 2.4 Particle Size Reduction

To assess the effect of mass transfer on conversion rates, full–dull textile‐grade PET pellets (Eastman) and smaller sized particles were employed as substrates. PET pellets (1800–2400 μm) were reduced in size using a commercial blender (Waring model CB15). Flakes or pellets were placed in the blender along with dry ice and run for 2‐min intervals for a total of 30 min. Dry ice was added as needed. The resulting material was separated using a series of sieving trays mounted on a motorized agitation tray (Humbolt Industries), and fractions in the range of 150–250 μm and 250–600 μm were collected for use.

## 3 Results and Discussion

**Scheme 1:**
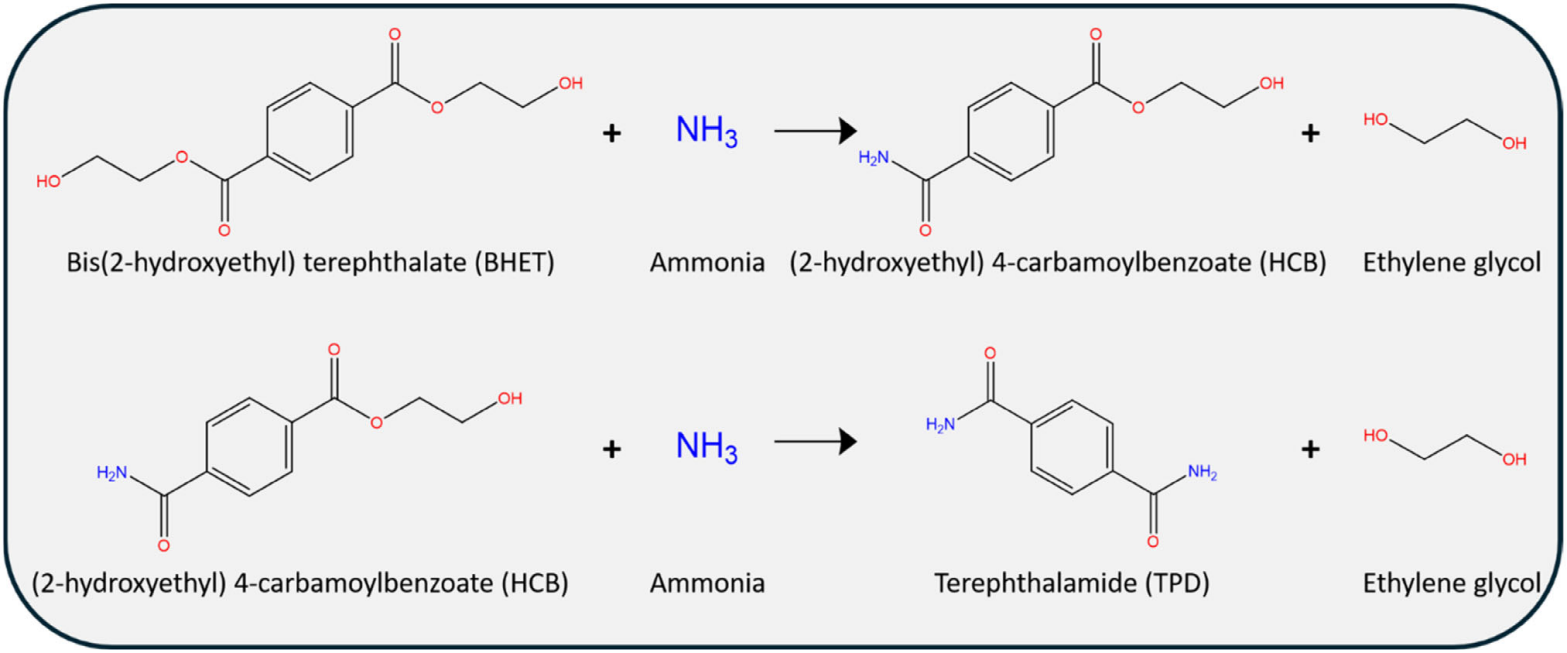
Ammonolysis of BHET. This is a two‐step reaction: 1) ammonolysis of one hydroxyethyl ester, followed by 2) ammonolysis of the other ester. Both reaction steps consume one molecule of ammonia and produce one mole of ethylene glycol.

The two‐step ammonolysis of BHET proceeds as illustrated in **Scheme** **1**. Initially, BHET reacts with ammonia to form HCB and EG. In the second step, the ammonolysis of the remaining ester group produces TPD and EG. Notably, it is found that the second reaction step is reversible in the presence of excess EG.

Before delving into the chemical kinetics, it is essential to examine the thermochemistry of the reactions depicted in Scheme 1. The equilibrium constant (*K*) is linked to the Gibbs free energy of reaction (*G*) through the fundamental relationship in Equation (1).

(1) $$\Delta G_{rxn}=-RTlnK$$

where *G* represents the Gibbs free energy, *R* is the ideal gas constant, and *T* is the absolute temperature. For a first approximation, the entropy of reaction can be disregarded. That is, the enthalpy of reaction can substitute for the free energy of reaction. The enthalpy of reaction can be approximated by calculating the difference between the sum of the bonds broken and the sum of the bonds formed. For the conversion of BHET to HCB, bond values from LibreTexts^[^ ^30^, ^31^ ^]^ yield an enthalpy of reaction equal to −23 kJ mol^−1^. Given that the same types and numbers of bonds are broken and formed, the conversion of HCB to TPD also results in an enthalpy of reaction equal to −23 kJ mol^−1^. These preliminary estimates suggest that the reaction is thermodynamically feasible. For a more precise estimation, neighboring electronic effects can be accounted for using group contribution methods. Following the procedure outlined in the supplementary materials based on L. Constantinou's work,^[^ ^31^ ^]^ the Gibbs free energy for each reaction step is calculated to be −33.9 kJ mol^−1^, corresponding to an equilibrium constant (K1) of 55,900. This strong indication of thermodynamic feasibility warrants a detailed investigation of the chemical kinetics.

The ammonolysis of esters has long been known to be catalyzed by EG.^[^ ^32^ ^]^ Previous studies typically show that the reaction rate no longer increases once a 3:1 molar ratio of EG to ester groups is reached.^[^ ^25^, ^33^ ^]^ EG is produced during the ammonolysis of PET, creating an autocatalytic effect. In this study, ammonolysis is carried out with an excess of EG, which simplifies the modeling and is most representative of how a large‐scale industrial process should be implemented.

Determining the start time of reactions is important in determining reaction kinetics. However, precisely pinpointing when *t* = 0 is challenging. There are two options widely used for estimating the start time of the reaction: 1) setting time to zero when the reactants are mixed, or 2) setting time to zero when the reaction reaches the setpoint temperature. In contrast to these practices, *t* = 0 is defined as the moment the reactor is placed in the thermal well and the temperature starts being recorded (which is only shortly after the reactants are mixed). Using this technique, the average temperature can be calculated from the recorded data. The techniques employed thus provide a high degree of accuracy in the reaction times and temperatures; details appear in the SI.

Determining the mixture composition upon halting the reaction also has challenges that must be addressed. BHET and the intermediate, HCB, are soluble in EG. These compounds are found in the liquid phase, even after cooling to 5 °C. For example, after reacting for 45 min at 50 °C, only 0.01 g (1 ppm) of the product is recovered as a solid. The mass fraction recovered as solids steadily increases with time as the reactant is converted to TPD. To determine the mole fractions during the course of the reaction, both recovered solids and liquids are analyzed via proton NMR. The recovered solids are predominantly TPD with some HCB present. The liquid phase is EG containing a dissolved mixture of BHET, HCB, and TPD.

**Figure 2:**
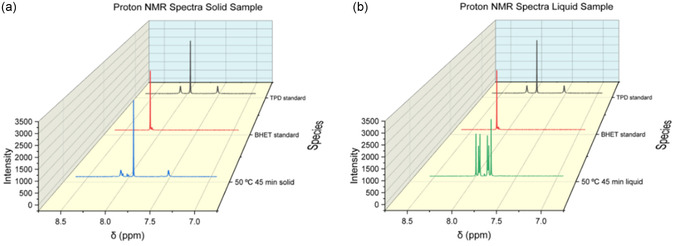
Proton NMR spectra. Left a) from back to front shows standards for TPD and BHET, and the recovered solid sample after 45 min of reaction at 50 °C. Right b) shows standards and the corresponding recovered liquid sample.

**Figure** **2** shows proton NMR results for standard samples of BHET and TPD used to help assign spectral peaks. The BHET standard showed impurity with a small multiplet (8.10–8.08 ppm). The results of the NMR assignments are as follows. TPD ^1^H NMR ((500 MHz, DMSO) *δ* 8.06 (s, 2H), 7.91 (s, 4H), 7.49 (s, 2H)), Bis(2hydroxyethyl) terephthalate ^1^H NMR ((500 MHz, DMSO) δ 8.12 (s, 4H), 4.97 (t, *J* = 5.7 Hz, 1 H),4.31(t, *J* = 4.9 Hz, 2H), 3.71(q, J = 5.7 Hz, J = 4.9 Hz, 2H)), and HCB (1137‐99‐1) ^1^H NMR ((500 MHz, DMSO) *δ* 8.14 (s, 1H), 8.07 – 8.02 (m, 2H), 8.00–7.95 (m, 2H), 7.57 (s, 1 H), 4.95(t, J = 5.7 Hz, 1 H), 4.31–4.25(m, 2H), 3.73–3.66 (m, 2H)). Non‐normalized NMR spectra of separate solid and liquid samples are provided in the SI.

**Figure 3:**
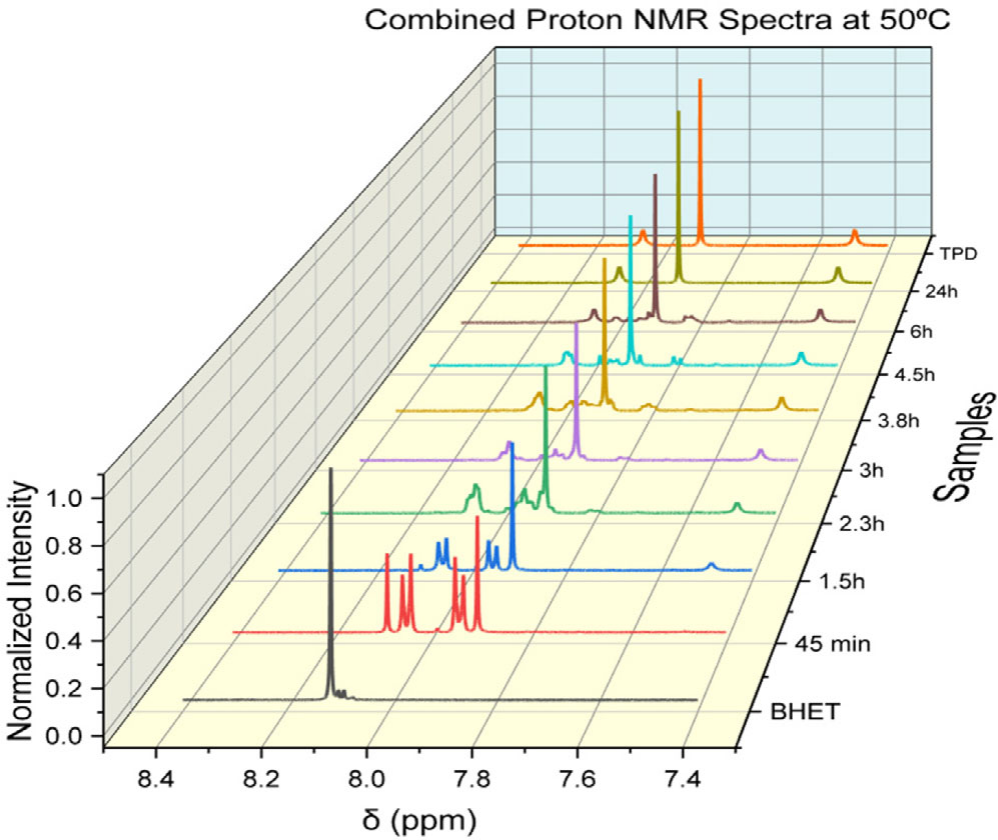
Combined NMR spectra from recovered liquid and solid samples showing the reaction trajectory from BHET through HCB to TPD.

**Figure** **3** shows the NMR spectra resulting from combining (adding together) the separate spectra from the recovered liquid and solid samples. The reaction trajectory is clearly observable, BHET is extinguished while the multiple indicating the presence of HCB grows and then diminishes. The peaks associated with TPD steadily increase. Using proton NMR spectra from both the recovered liquid and solid phases, a material balance determines the mole fractions of each component at every reaction time.

Mole fractions may not be the appropriate basis for a chemical kinetics model. An activity‐based model may be required when a mixture is nonideal. To investigate this issue, activity coefficients are calculated using different thermodynamic models implemented within the ASPEN Plus^TM^ software environment.^[^ ^34^ ^]^ Three thermodynamic models are employed, namely, PC‐SAFT,^[^ ^35^, ^36^ ^]^ Uniquac,^[^ ^37^ ^]^ and NRTL.^[^ ^38^ ^]^ Uniquac and NRTL, estimate the activity coefficients to be about 0.8 for ammonia; the PC‐SAFT model returns disparate values considered to be anomalous due to a lack of appropriate data. When fitting the kinetics data, correction of the mole fractions using activity coefficients resulted in similar “goodness of fit” metrics. The SI contains a more detailed discussion of activity coefficients and presents results of the thermodynamic modeling. Based on the activity calculations, the kinetic model is developed using mole fractions.

A chemical kinetics model is developed based on mole fractions and further simplifying assumptions. The rate of change of ammonia is expressed in Equation (2).

(2) $$\frac{dx_{AMM}}{dt}=-k_{1}x_{EG}x_{AMM}x_{BHET}-k_{2}x_{EG}x_{AMM}x_{HCB}$$

In Equation. (2) $x_{EG},\;x_{AMM}$, $x_{BHET}$, and $x_{HCB}$ are the mole fractions of EG, ammonia, BHET, and HCB; $k_{1}$ and $k_{2}$ are the reaction rate constants for the first and second reaction steps, respectively. Due to its catalytic effect, the concentration of the EG is included. But due to it being present in great excess, far above the 6:1 molar ratio that saturates the catalytic effect, it is taken as constant (even though a small amount is generated during the reaction). Similarly, ammonia is in excess and is also taken to be constant. These well‐justified assumptions enable both reaction steps to be treated as pseudo‐first‐order reactions; the constant concentrations are lumped into the rate constants. The rates of reaction may be expressed using Equation. ((3), (4))–(5).

(3) $$\frac{dx_{BHET}}{dt}=-k_{1}x_{EG}x_{AMM}x_{BHET}=-k_{1}^{\prime }x_{BHET}$$

(4) $$\frac{dx_{HCB}}{dt}=k_{1}x_{EG}x_{MM}x_{BHET}-k_{2}x_{EG}x_{AMM}x_{HCB}=k_{1}^{\prime }x_{BHET}-k_{2}^{\prime }x_{HCB}$$

(5) $$\frac{dx_{TPD}}{dt}=k_{2}x_{EG}x_{AMM}x_{HCB}=k_{2}^{\prime }x_{HCB}$$

where $x_{TPD}$ is the mole fraction of TPD and $k_{1}^{\prime }$ and $k_{2}^{\prime }$ are the pseudo‐first‐order rate constants for the first and second reaction steps, respectively.

Equation. ((3), (4))–(5) were solved using Laplace transforms (see SI for further details), resulting in Equation ((6), (7))–(8).

(6) $$x_{BHET}\left(t\right)=x_{BHET,0}exp\left(-k_{1}^{\prime }t\right)$$

(7) $$x_{HCB}\left(t\right)=Y\exp\left(-k_{1}^{\prime }t\right)-Y\exp\left(-k_{2}^{\prime }t\right)$$

(8) $$x_{TPD}\left(t\right)=\;x_{BHET,0}-Z\exp\left(-k_{1}^{\prime }t\right)+Y\exp\left(-k_{2}^{\prime }t\right)$$

Here $x_{BHET,0}$ is the mole fraction of BHET at time t = 0 and *Y*, *Z* are defined by Equation (9)–(10).

(9) $$Y=\frac{x_{BHET,0}k_{1}^{\prime }}{k_{2}^{\prime }-k_{1}^{\prime }}$$

(10) $$Z=\frac{x_{BHET,0}k_{2}^{\prime }}{k_{2}^{\prime }-k_{1}^{\prime }}$$

Equation ((6), (7), (8), (9))–(10) describe the reaction pathway as a function of time at a constant temperature.

To include the effects of temperature, Equation ((6), (7), (8), (9))–(10) are combined with the Arrhenius equation $\left(k=A\exp\left(-\frac{E_{a}}{RT}\right)\right)$ describing the temperature dependence of the rate constants. This combination provides a mathematical description of the mole fraction of each species as a function of time and temperature. The mole fractions versus time data, for all reaction temperatures, are simultaneously fit to the model using the curve fit function from scipy.Optimize library of the Python computer programming language. The fitting routine optimizes the “goodness of fit” and returns the activation energies and pre‐exponential factors for the rate constants. Pre‐exponential factors of *A*_1_ = h^−^^1^ and *A*_2_ = h^−^^1^ are found for the first and second steps of the reaction, and the activation energies are *E*_*a*1_ = kJ mol^−1^ and *Ea*_2_ = kJ mol^−1^.

**Figure 4:**
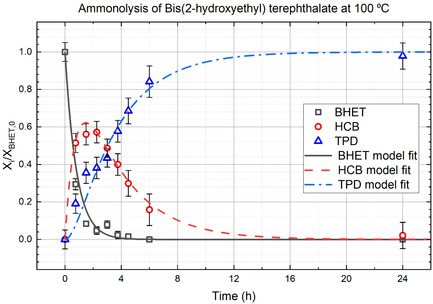
Model fit of mole fractions data for reaction at 100 °C. BHET is consumed throughout the reaction, HCB is first generated and then consumed, and the final product is TPD, obtained at yields of 85–95% of theoretical.

Measured mole fractions as a function of time at 100 °C are plotted alongside the model fit in **Figure** **4**. Overall, the model provides a satisfactory fit to the data with *R* ^2^ values of 0.965, 0.988, and 0.917 for curves of BHET, HCB, and TPD, respectively. The mole fraction data and model fit for the ammonolysis of BHET at temperatures of 50 °C, 75 °C, and 125 °C can be found in the SI.

**Figure 5:**
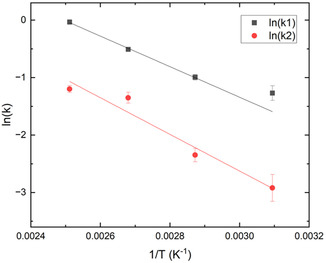
Arrhenius plot of k1 and k2 against inverse temperature.

An Arrhenius plot is provided in **Figure** **5**. The lines represent calculated values using the best‐fit values of the pre‐exponential factors and activation energies. Points with error bars are provided to show the range of expected values based on the error associated with the fitting procedure. The activation energies fall within a reasonable range for the ammonolysis of esters.^[^ ^33^ ^]^ The relatively low activation energy for the first reaction step may be attributed to two factors: 1) reduced electron density due to the proximity of the hydroxy group to the ester, and 2) the catalytic effect of EG. The collision factor *A*_2_ is approximately one and a half times greater than *A*_1_; this indicates that, compared to BHET, the frequency of collisions with ammonia leading to reaction is higher for HCB. Factors contributing to this increased collision frequency include bond angles, molecular size (reaction cross section), and diffusivity. Given the large difference in the pre‐exponential factors, the various contributing factors are examined in greater detail.

Bond angle differences are investigated using molecular dynamics in the ChemDraw 3D software package. Conformational energies are minimized; resulting structures are shown in the SI. Bond angles near the ester bond do not change within a 0.1° tolerance. Given these small differences, it is unlikely that bond angle changes play a significant role in increasing the collision factor.

The molecular cross section (*σ*_AB_) can be thought of as the average distance between species when they collide and is directly proportional to the collision frequency. The decrease in molecular weight implies a decreased molecular cross section, which would mean a decreased collisional frequency. Interpretation is subtle, though, because while molecular collisions are reduced, the same is not necessarily true for the ester group itself, because it retains the same size.

A factor that does help explain the difference is diffusivity. In solute‐solvent reactions, the collision factor is directly proportional to the solute's diffusivity, which depends on its molecular weight. In the first reaction step, the reactant's molecular weight decreases from 254.24 g mol^−1^ to 209.20 g mol^−1^, an 18% reduction. This decrease impacts the diffusivity and, consequently, the collision rate. Graham's Law^[^ ^39^, ^40^ ^]^ can be used to approximate how this reduction in molecular weight affects the diffusivity, as shown in Equation (11).

(11) $$D\propto \frac{1}{\sqrt{M_{w}}}$$

In Equation (11), D is the diffusivity, and M_***w***_ is the molecular weight of the solute. The relative change in diffusivity based on the change in molecular weight is calculated in Equation (12).

(12) $$\frac{D_{HCB}}{D_{BHET}}\propto \frac{\sqrt{M_{W,BHET}}}{\sqrt{M_{w,HCB}}}=\frac{\sqrt{254.24}}{\sqrt{209.20}}=1.102$$

where $D_{HCB}$, and $D_{BHET}$ are the diffusivities of HCB and BHET, and $M_{W,HCB}$ and $M_{W,HET}$ are the molecular weights of HCB and BHET, respectively. The result shows a modest 10.2% increase in diffusivity. While consistent with experimental observations, changes in diffusivity cannot fully explain the differences in collision factors.

It is notable that the present system is more complex than what might be expected due to hydrogen bonding effects. Indeed, earlier studies on the catalytic effects of EG and other glycols like glycerol invoke the idea that the glycols form hydrogen bonding networks with the carbonyl groups of the esters. A full explanation of the orientation effects of hydrogen bonding in the system is beyond the scope of the present study.

Activation energies for the two reaction steps are justifiable using conventional molecular reasoning. Differences in activation energies are conventionally attributed to a combination of steric and electronic structure effects. For example, ester groups are more electron withdrawing than amide groups.^[^ ^41^ ^]^ In BHET, two opposing esters within the structure exert an even pull on electrons. Conversely, HCB contains one ester and one amide. The ester's stronger electron‐withdrawing effect surpasses that of the opposing amide group. Consequently, the ester bond in HCB possesses higher electron density than those in BHET. As a result, the nucleophilic attack in the second reaction step requires a higher activation energy to break the ester bond. That is, the second carbonyl carbon is more electronegative and thus less susceptible to nucleophilic attack, thereby necessitating a greater activation energy.

### 3.1 Ammonolysis of Post‐Consumer PET Waste

To evaluate the practical utility of ammonolysis as a viable upcycling technology, waste PET thermoformed containers were collected from the campus recycling center. These containers, typically of the “clamshell” type, were hand washed and cut into flakes. Some flakes were granulated into small particles. These differently sized particles were used as substrates for ammonolysis in glycol. The objective in using different‐sized particles is to evaluate the significance of mass transfer limitations; when such diffusional restrictions are present, the rate of reaction is no longer the rate‐limiting step.

**Figure 6:**
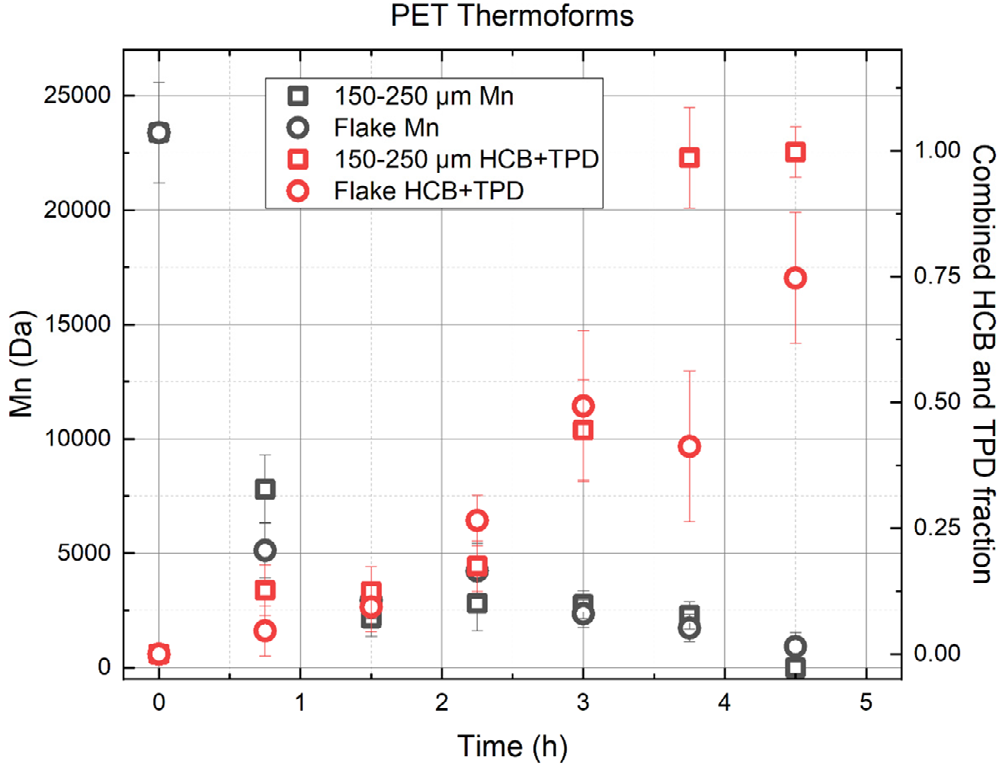
The number average molecular weight (Mn) as a function of time during the ammonolysis of thermoform PET. The flake from a post‐consumer clamshell cut into 1 cm^2^ squares degrades at a very similar rate to ground material composed of particles sized between 150–250 μm. These similar conversion rates indicate little benefit of particle size reduction of PET clamshells beyond a simple shredding process.

**Figure** **6** shows that shredded flakes and smaller granulated materials exhibit similar depolymerization rates. Importantly, under these modest conditions, both substrates are completely converted within 6 h. Molecular weight data suggests little difference in depolymerization rates. However, the amounts of TPD and HCB generated show that the granulated material was nearly fully converted at 3.8 h, whereas the flake was only about half converted. During the initial hour of reaction, the molecular weight decreases sharply. Subsequently, it plateaus and changes very slowly. This behavior is expected based on the theory of step‐growth (i.e., condensation) polymerization. That is, when forming a high polymer, it is at the latest stages of polymerization that the molecular weight increases rapidly. Here, the reverse is observed, consistent with a random chain scission mechanism.

The data demonstrate that there is very little incentive to perform particle size reduction of waste PET thermoforms when conducting ammonolysis by the present methodology. Conversion rates are similar for both flakes and smaller granulated particles. Notably, the typical thermoform wall thickness is about 400 μm, so its minimum dimension is similar (same order of magnitude) as the 150–250 μm particles. Accordingly, the geometric differences between the planar flakes and semi‐spherical particles are negligible. Additionally, it is observed that after 45 min of reaction, the flake becomes frangible (presumably due to the decrease in molecular weight) and easily breaks into a powder. Given the constant agitation in the reactor, it is reasonable to hypothesize that particle size reduction occurs in situ during the course of the reaction. The added costs associated with energy‐intensive milling of PET to micron‐size particles are presumably not justified by the slight increase in reaction rates.

### 3.2 Ammonolysis of PET of Varying Sizes

As the particle size is increased further, mass transfer limitations become very important. Figure 6 shows decreased conversion rates when PET pellets ranging from 1800–2500 μm are used as the substrate. Ammonolysis to HCB and TPD, indicating full depolymerization, of 150–250 μm particles requires 5 h, for 250–600 μm particles it takes 18 h, and for 1800–2500 μm 96 h are needed! This very significant variation in conversion rates based on particle size is due to mass transfer limitations.

Depolymerization of PET can be analyzed in terms of the number of ester bonds cleaved. As demonstrated in the ammonolysis of BHET, there are two ester types: the ester bond of BHET (ester type one, ET1) and the ester bond of HCB (ester type two, ET2). ET1 bonds have no adjacent amide groups, while ET2 bonds have an amide bond in the *para* position. There are differences between the behavior of these esters; the rate of ammonolysis of ET1 ($k_{1}^{\prime }$ = 0.958 ± 0.076 h^−1^) is greater than the rate for ET2 ($k_{2}^{\prime }$ = 0.359 ± 0.034 h^−1^). Ammonolysis of the ester bond in PET is molecularly similar to ammonolysis of ET1 in BHET. Backbone ester linkages in PET are not near an amide bond. Therefore, measuring the disappearance of esters in PET can be modeled by the disappearance of ET1 bonds.

The disappearance of ET1 bonds can be expressed by a pseudo‐first‐order kinetic rate law. To do so, $x_{EG}$ and $x_{AMM}$ are treated as constants due to their large excess. This justified assumption leads to Equation (13).

(13) $$\frac{dx_{ET1}}{dt}=-kx_{EG}x_{AMM}x_{ET1}=-k\prime x_{ET1}$$

Here $x_{ET1}$ is the mole fraction of ET1 bonds, *k* is the apparent rate constant, and k′ is the apparent pseudo–first‐order rate constant. The mole fraction of ET1 bonds is defined by Equation (14).

(14) $$x_{ET1}=\frac{n_{ET1}}{n_{ET1}+n_{ET2}+n_{Amide}+n_{AMM}+n_{EG}}$$

In Equation (14), $n_{ET1}$, $n_{ET2}$, $n_{Amide}$, $n_{AMM}$, and $n_{EG}$ are the number of moles of ET1 bonds, ET2 bonds, amide bonds, ammonia, and EG, respectively. The differential equation is solved to find Equation (15)

(15) $$x_{ET1}\left(t\right)=x_{ET1,0}exp\left(-k\prime t\right)$$

where $x_{ET1,0}$ is the initial mole fraction of ET1 bonds.

Application of Equation (15) requires calculation of the mole fraction of ester bonds in the PET substrates. The number of moles of ET1 bonds can be calculated by assuming every PET repeat unit has two ET1 bonds. PET with an amide end group would have two less ET1 bonds due to the existence of the amide and corresponding ET2 bond. However, the small number of chain ends during the early stages of depolymerization means this molecular detail can be safely ignored. Accordingly, the number of ET1 bonds is calculated by dividing the number average molecular weight (Mn) of the polymer by the molecular weight of a repeat unit. The resulting number of repeat units is multiplied by two to find the number of ET1 bonds. The mole fraction of ET1 calculated by Equation 14 is fit to Equation 15 to determine the apparent pseudo‐first‐order rate constants for each particle size.

**Figure 7:**
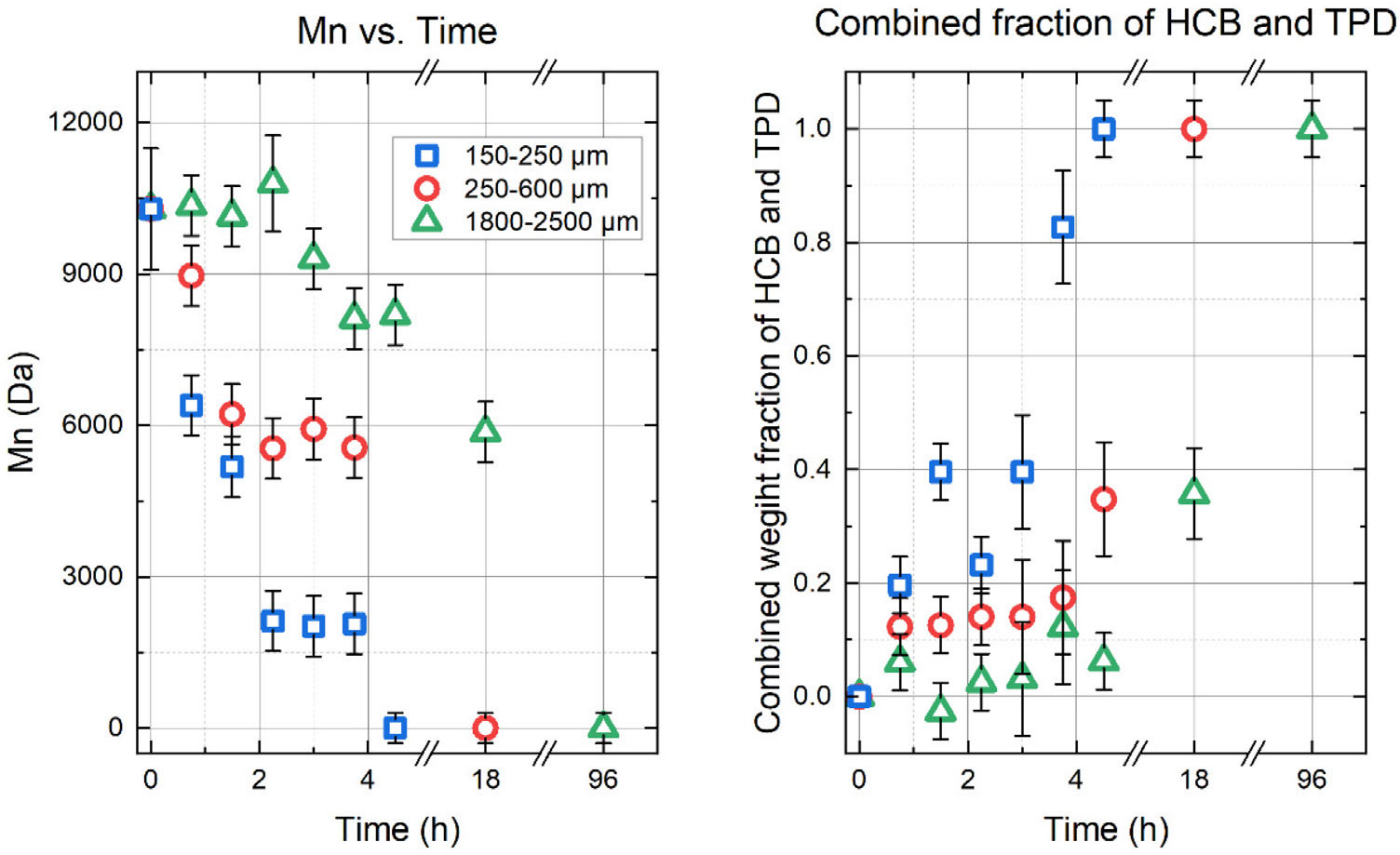
(Left) Number average molecular weight (Mn) versus time for varying PET particle sizes during the first 5 h of reaction. (Right) When the reactions were run to completion as measured by conversion to TPD, the 150–250 μm particles took 4.5 h, the 250–600 μm took 18 h, and the 1800–2500 μm particles after 96 h.

**Figure 8:**
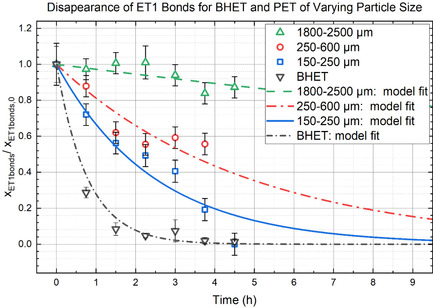
The disappearance of ET1 bonds for various particle sizes of PET and their model fit compared to that of BHET. ET1 bonds are ester bonds similar to those of BHET, in the *para* position to other ester bonds.

**Figure** **7** shows the disappearance of ET1 bonds for BHET and PET particles of different sizes at 100 °C. The goodness of fit *R* ^2^ values are 0.968, 0.974, 0.930, and 0.959 for BHET, 150–250 μm particles, 250–600 μm particles, and 1800‐2500 μm pellets, respectively. The apparent reaction rate constants are $k_{150-250}^{\prime }$=0.350 ± 0.033 h^−1^, $k_{250-600}^{\prime }$=0.110 ± 0.017 h^−1^, and $k_{1800-2500}^{\prime }$ = 0.025 ± 0.003 h^−1^. This observed trend of decreasing apparent rate of reaction with increasing particle size is consistent with mass transfer being the rate‐limiting step **Figure** **8**.

The ester conversion rates of PET were compared to the ammonolysis rate of BHET at 100 °C to assess the effects of mass transfer limitations. The mole fraction of ET1 bonds in BHET is expressed in terms of the moles of BHET ($n_{BHET}$), HCB ($n_{HCB})$, and TPD ($n_{TPD})$in Equation (16).

(16) $$x_{ET1,BHET}=\frac{2\left(n_{BHET}\right)}{2\left(n_{BHET}+n_{HCB}+n_{TPD}\right)+n_{AMM}+n_{EG}}$$

An effectiveness (η) factor is defined using Equation (17).

(17) $$\eta =\frac{rate\;of\;disapearance\;of\;ET1\;bonds\;in\;PET}{rate\;of\;disapearance\;of\;ET1\;bonds\;in\;BHET}=\frac{-k^{\prime }x_{ET1}}{-k_{1}^{\prime }x_{ET1,BHET}}$$

Looking at the initial rate of reaction, where t=0, the effectiveness equation is simplified in Equation (18).

(18) $$\eta =\frac{k^{\prime }x_{ET1,0}}{k_{1}^{\prime }x_{ET1,BHET,0}}$$

Here $x_{ET1,BHET,0}$ is the initial mole fraction of ET1 bonds in the BHET reaction. The corresponding Thiele modulus (∅) is given by Equation (19).^[^ ^42^, ^43^ ^]^

(19) $$\phi =L*\sqrt{\frac{k}{D_{eff}}*\frac{n+1}{2}*C_{s}^{n-1}}$$

where $L=\frac{Volume}{Srface\;Area}$, k is the apparent rate constant for the disappearance of ET1 esters in the PET sample, $D_{eff}$ is the effective diffusion coefficient of ammonia in PET, n is the reaction order, and *C*_s_ is the concentration at the surface. Since the reaction order is one, the surface concentration is lost from the equation. The general relationship between the effectiveness factor and the Thiele modulus for spheres is expressed by Equation (20).

(20) $$\eta =\frac{3}{\phi^{2}}\left(\phi \coth\left(\phi \right)-1\right)$$

The observed conversion data can be fit to the form of Equation (18) using the effective diffusivity of ammonia in PET as a fitting parameter.

**Figure 9:**
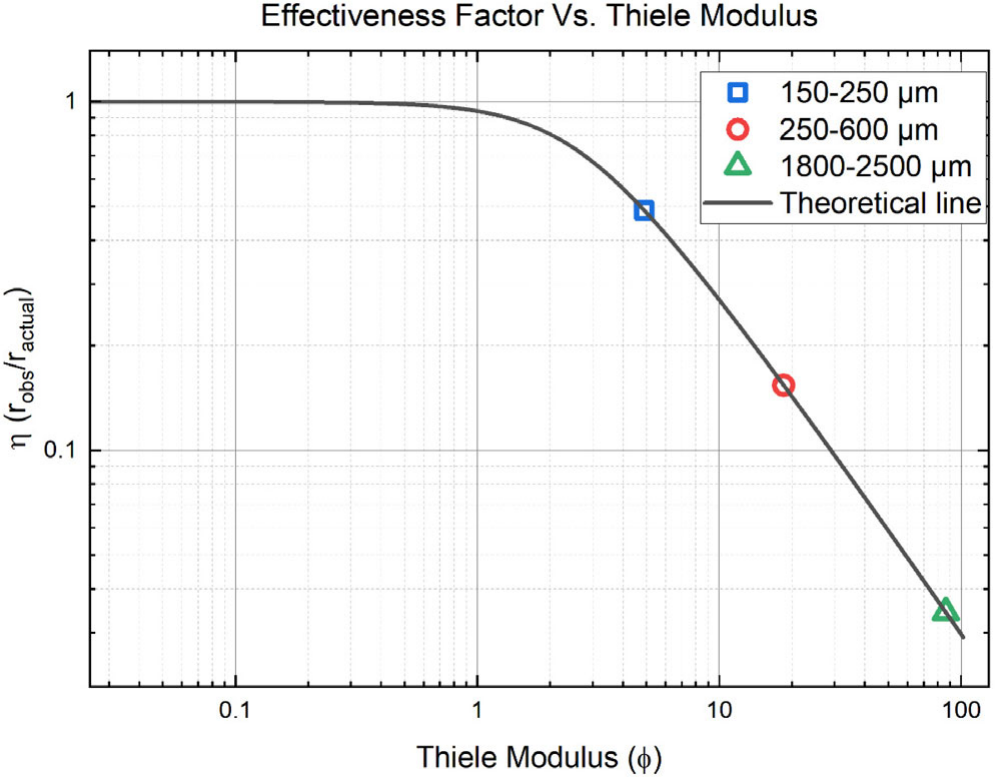
Effectiveness factor versus Thiele modulus. The effectiveness factor is calculated by dividing the apparent rate of ammonolysis of PET at a given particle size by the reaction rate for BHET. The diffusivity of ammonia in PET serves as the single fitting parameter to match the data to the theoretical line. The resulting diffusivity value of 1.37 ± 0.48 E‐7 cm^2^ s^−1^ is in good agreement with independent literature values.

By fitting Equation (20) to the calculated effectiveness factors, a Thiele modulus is found. Equation (19) then yields an effective diffusivity. The volume‐to‐surface area is taken as that for spherical particles with a diameter equal to the mean particle size. **Figure** **9** shows the effectiveness factor versus Thiele modulus fit. Numerically solving for $D_{eff}$ using Equation. (19) yields a diffusivity for each particle size. The average effective diffusivity of ammonia in PET over the three different particle sizes is found to be 1.37 ± 0.48 E‐7 cm^2^ s^−1^.

There are many studies on the diffusion of different molecules in PET.^[^ ^44^ ^]^ However, no specific study on the diffusivity of ammonia in PET at elevated temperatures appears in the literature. However, one study found that the diffusion coefficient of water in amorphous PET at 100 °C is 1.35 E‐6 cm^2^ s^−1^.^[^ ^45^, ^46^ ^]^ Interestingly, this value is one order of magnitude higher than the determined diffusion coefficient of the present study. The difference can be attributed to PET crystallinity and the size of the solvent molecules. The PET in the present study is semi‐crystalline, but the PET used in previous studies was 100% amorphous. The diffusion in amorphous PET is faster than in crystalline regions. In addition, water is smaller than ammonia. By approximating molecular volumes as the sum of each atom's volume, ammonia has a molecular volume of ≈26.3 Å^3^, while water has one of 19.8 Å^3^. In general, smaller molecules diffuse more rapidly than larger ones. Therefore, despite the disparity, these two results are compatible.

Another way to analyze the diffusivity coefficient is to compare it with reported values for the diffusivity of ammonia in other polymers.^[^ ^47^, ^48^ ^]^ Comparing low‐density polyethylene and polydimethylsiloxane with various fluoropolymers, Makhloufi al. reported diffusivities ranging from 5.9 E‐8 cm^2^ s^−1^ to 4.18 E‐6 cm^2^ s^−1^ at 21 °C.^[^ ^49^ ^]^ Accordingly, the value determined in this study (1.37 ± 0.48 E‐7 cm^2^ s^−1^) falls within the range of known ammonia diffusivities in other polymers at temperatures above their glass transition temperatures.

While published literature lacks specific information on the diffusion of ammonia in PET, models can provide estimates for comparison. Ewender developed a function based on molecule size and temperature to predict diffusion in PET.^[^ ^50^, ^51^ ^]^ Using this model, the calculated diffusion coefficient for ammonia in PET is 3.18 E‐8 cm^2^ s^−1^, this value is approximately six times lower than the value determined in our study (1.37 ± 0.48 E‐7 cm^2^ s^−1^).

The discrepancy in diffusion values between various sources has a few contributing factors. First, the value determined here is an “effective” diffusivity in a multicomponent molecular soup of reactants and products. Additionally, excess EG is present, which swells the PET and makes it more liquid like and thus leads to a larger rate of diffusion when compared to a homogeneous polymer phase. For example, Zheng investigated PET swelling in excess EG,^[^ ^52^ ^]^ observing increased end‐to‐end distance and radius of gyration via molecular dynamics simulations.^[^ ^53^ ^]^ Accordingly, discrepancies with the Ewender model are attributed to swelling of PET.

## 4 Conclusions

Details of the experimental ammonolysis of PET in excess EG are reported for the first time. Using BHET as a model compound, the rate constants at 100 °C for sequential ammonolysis of the two ester bonds are $k_{1}^{\prime }$ = 0.958 ± 0.076 h^−1^ and $k_{2}^{\prime }$ = 0.359 ± 0.034 h^−1^. Activation energies for the two reaction steps are *E*_*a*1_ = 7.01 ± 0.65 kJ mol^−1^ and *Ea*_2_ = 13.74 ± 0.660 kJ mol^−1^ and the corresponding collision factors are *A*_1_ = 97.2 ± 11.4 h^−1^ and *A*_2_  = 318.9 ± 35.7 h^−1^.

Ammonolysis experiments on 1 cm^2^ squares (flakes) and 150–250 μm granulated particles produced similar conversion rates. Accordingly, for ammonolysis, simple shredding of PET thermoforms is sufficient to avoid significant diffusional limitations. Unlike enzymatic and other processes based on surface reactions, no further reduction in particle size is needed.

The present study demonstrates the profound importance of understanding diffusional limitations. For particles of only 1 millimeter in average thickness, the rate of reaction is decreased by a factor of ten! Experiments performed on PET particles having different average sizes (150–250 μm, 250–600 μm, 1800‐2500 μm) allow the construction of an effectiveness factor. Using the classical approach enables calculation of a Thiele modulus from which the effective diffusivity of ammonia in PET can be calculated. At 100 °C, this value is estimated to be 1.37 ± 0.48 E‐7 cm^2^ s^−1^. This effective diffusion coefficient incorporates the effects of excess EG, which swells the polymer and enhances diffusion rates.

Understanding ammonolysis conversion rates across different PET particle sizes informs efficient process design and facilitates process scale‐up. The comprehensive understanding of the ammonolysis of PET in EG provided can play an important role in improving material reuse and fostering a more circular economy.

## Conflict of Interest

The authors declare no conflict of interest.

## Supporting information

Supplementary Material

## Data Availability

The data that support the findings of this study are available in the supplementary material of this article.
