# Supplementary material for: Upcycling of Waste Poly(ethylene Terephthalate): Ammonolysis Kinetics of Model Bis(2‐Hydroxyethyl Terephthalate) and Particle Size Effects in Polymeric Substrates
Source: ChemSusChem. 2025 Jun 30;18(15):e202500509. doi: 10.1002/cssc.202500509 (PMC12302304; doi:10.1002/cssc.202500509)
Supplement: Supplementary file 1 — Supplementary Material [file CSSC-18-e202500509-s001.pdf]

Supplemental Materials for *Upcycling of zero-valued poly(ethylene terephthalate) II: Ammonolysis kinetics of model bis(hydroxy-ethyleneterephthalate) and particle size effects in polymeric substrates*

Contents

1.0 Mole fractions from NMR 5

1.1 Example of determining mole fractions 9

2.0 Differential equations solved 17

3.0 Supplemental figures 22

3.1 Parr multi-reactor system .... **Error! Bookmark not defined.**

3.2 P-xy figures .... **Error! Bookmark not defined.**

3.3 Temperature ramp figures 24

3.4 NMR figures 25

3.4.1 BHET 50°C proton NMRs 25

3.4.2 BHET 75°C proton NMRs 28

3.4.3 BHET 100°C proton NMRs 31

3.4.4 BHET 50°C proton NMRs 34

3.4.5 PET of Varying size- Proton NMRs 37

3.4.6 PET Thermoform proton NMRs 41

3.5 Model fit figures 22

3.6 Activity model figure 45

3.7 Crystallinity vs. Time 45

3.8 Disappearance of ester bonds .... **Error! Bookmark not defined.**

1.0 Reaction Procedures

1.1 Reactor Cleaning and Assembly

1. The reactor components are shown in Figure S1.
2. Prepare a solution of Alconox or other detergent solution according to the manufacturer's recommendations
3. Place the quartz insert into a quart size jar and cover with the cleaning solution (see Figure S2)
4. Place the reactor cylinder and head (with thermowell attached) into a metal canister of approximately quart size and cover with cleaning solution (see Figure S2)
5. Place the containers in the ultrasound bath and sonicate for 30 minutes (see Figure S2)
6. Rinse metallic components with acetone and the quartz with isopropyl alcohol, place in a dust and contaminant free environment to dry.

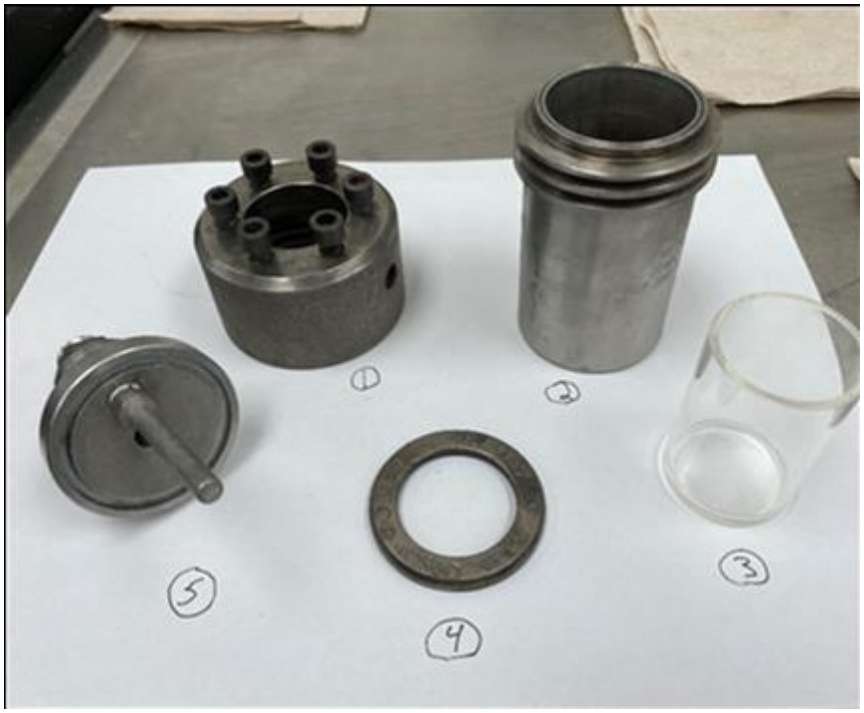

Figure S1. Reactor components (1. Collar with retention bolts, 2. Cylinder, 3. Quartz insert, 4. Retention ring, 5. Head with thermowell).

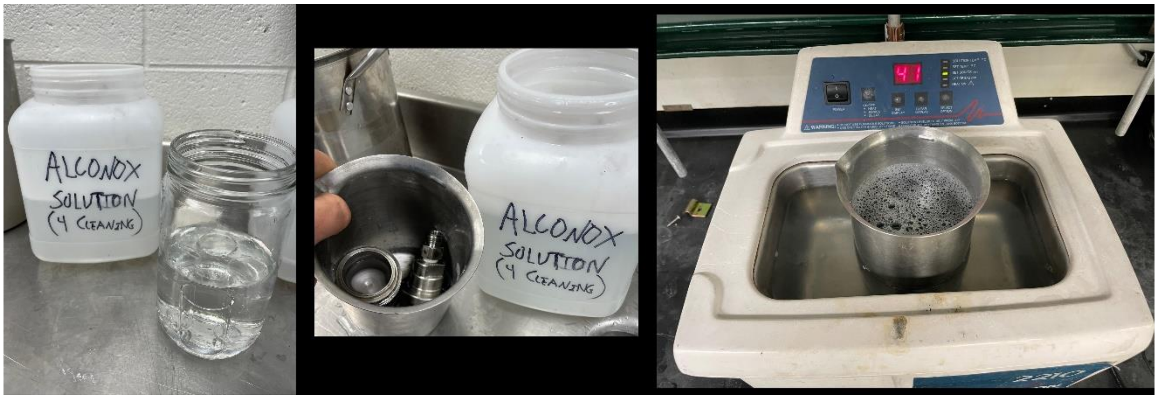

Figure S2. Cleaning of reaction contacting reactor components.

1.2 Loading the reactants

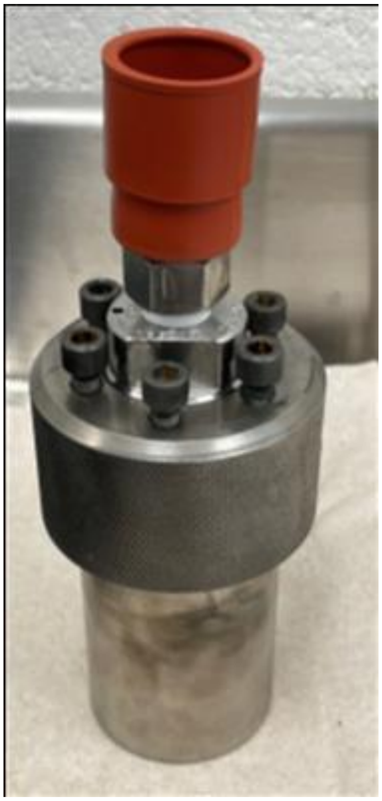

Figure S3. Assembled reactor, containing solids, purged with Argon gas, and fitted with a rubber septum to enable liquid charging using a syringe.

1. Charge 1-5 grams of solids or non-volatile liquids into the quartz liner and blanket with Argon gas.
2. Assemble the reactor, purge again with Argon gas and seal with rubber septum (see Figure S3). During assembly ensure the PTFE gasket is in place on the reactor head, that the head bolts are backed out, and that the compression ring is placed between the reactor head and collar bolts. Hand tighten the collar over the cylinder. Seal the reactor by tightening the hex bolts using a pattern that crosses over the center as each bolt is tightened to prevent misalignment.
3. Using a syringe, load the reactor vessel with 15-10 ml of liquid reactants (ammonia in methanol and ethylene glycol)
4. Place the reactor into the Parr 5000 multi-reactor system; make sure it is aligned in the heating mantle. **Be sure to connect the correct thermocouple and gas inlet for the corresponding well location.** Remove the rubber septum and connect the gas inlet; finger tighten the connection using a progressive forwards then backwards then forwards tightening to ensure no cross-threading. Simple finger tightening is sufficient.
5. Check that the nitrogen tank is open and that the regulator is adjusted to the desired pressure (typically 1200 psia when reactor reached temperature) and that the vent valve on the manifold (the lower valve) is closed. Open the gas inlet valve (the upper valve on the Parr 5000 manifold) to allow the reactor to pressurize. Watch the pressure gauge for the corresponding reactor presented on the computer. When the reactor reaches the pressure of the nitrogen tank regulator, close the reactor inlet valve.
6. Wait for 1-2 minutes and note any changes in pressure. If the gauge pressure is unchanged or drops very little (a couple of psig) then proceed to the next step. Otherwise, slowly vent the remaining pressure, disassemble the reactor, and start the procedure over.
7. Turn on the corresponding temperature controller and enter the desired setpoint; manually adjust the agitation speed to the desired value (5-8 hundred RPM).
8. During the reaction, use the PC software will monitor and record the temperature, pressure and speed of rotation. Values may be observed in either the controller window or as a real time strip-chart type plot in the charting window.

1.3 Thermally quenching the reaction

1. At the end of the desired reaction time, hit the large "STOP" button beneath the corresponding controller. Using heavy, heat resistant gloves, remove the reactor from the thermal well and place it in an ice bath contained by a metal pot (see Figure S4).

2. When the thermocouple reads 5°C, slowly release the pressure by opening the gas release valve (the lower valve on the manifold). Always wear heat resistant gloves, a flame and chemical resistant lab coat, and safety glasses - keep the hood sash as closed as possible.
3. Disconnect the reactor from the gas inlet and reattach rubber septum to avoid sample contamination. Dry the outside of the reactor with cloth or paper towels.
4. Carefully disassemble the reactor. Note: because the reactor is now cold and the metallic components have shrunk, disassembly should be easy. If fittings are tight, double check your procedures. Bolts may be loosened using the vice and a wrench, but final disassembly should be by hand and in a chemical hood.
5. Remove the glass insert and drain any liquids remaining in the metal cylinder into a sample collection container. Retain both linear and spillover for subsequent product work up. Remove the other reactor components from the hood and place out of the way for subsequent cleaning.

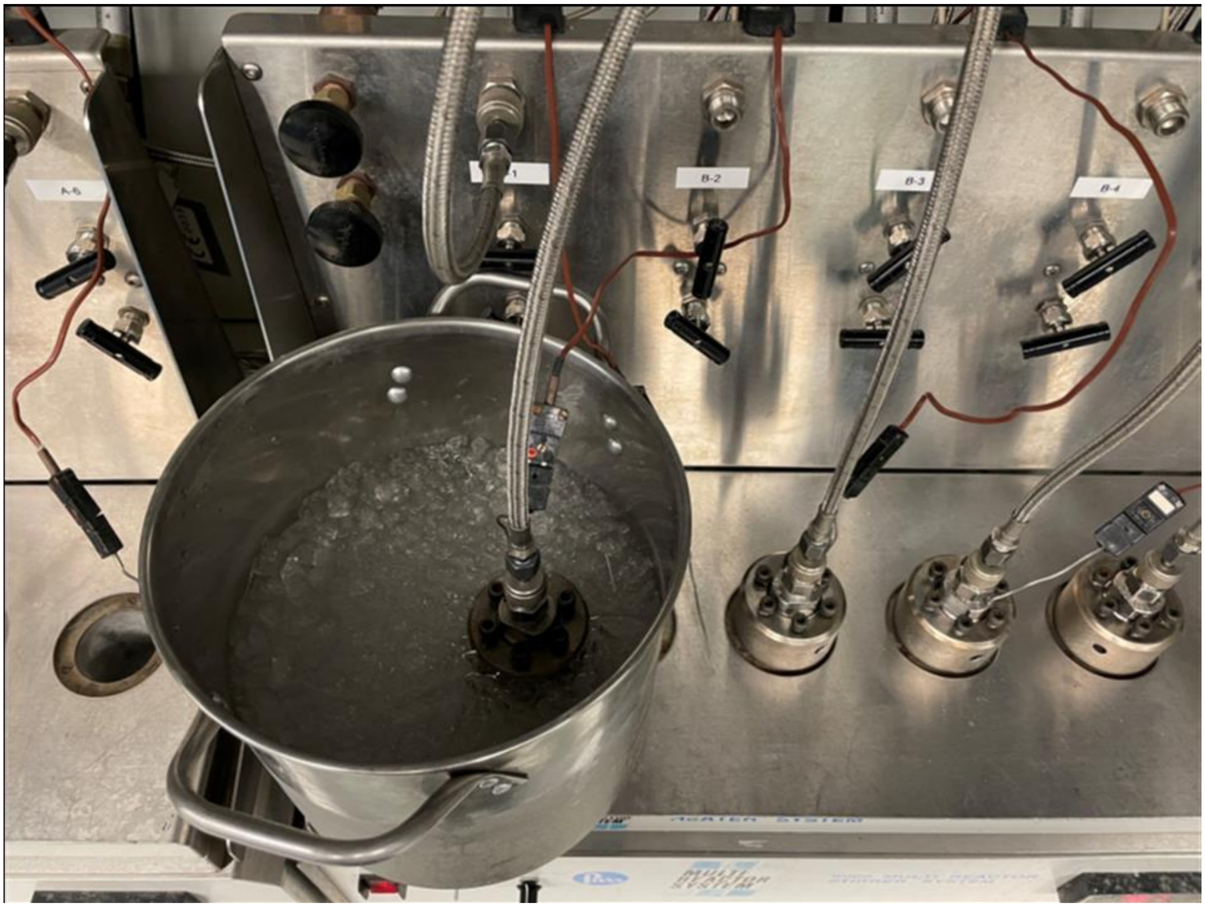

Figure S4. Reaction ended by ice bath quenching. Note: do not quench until thermocouple reads 100°C or less.

2.0 Mole fractions from NMR

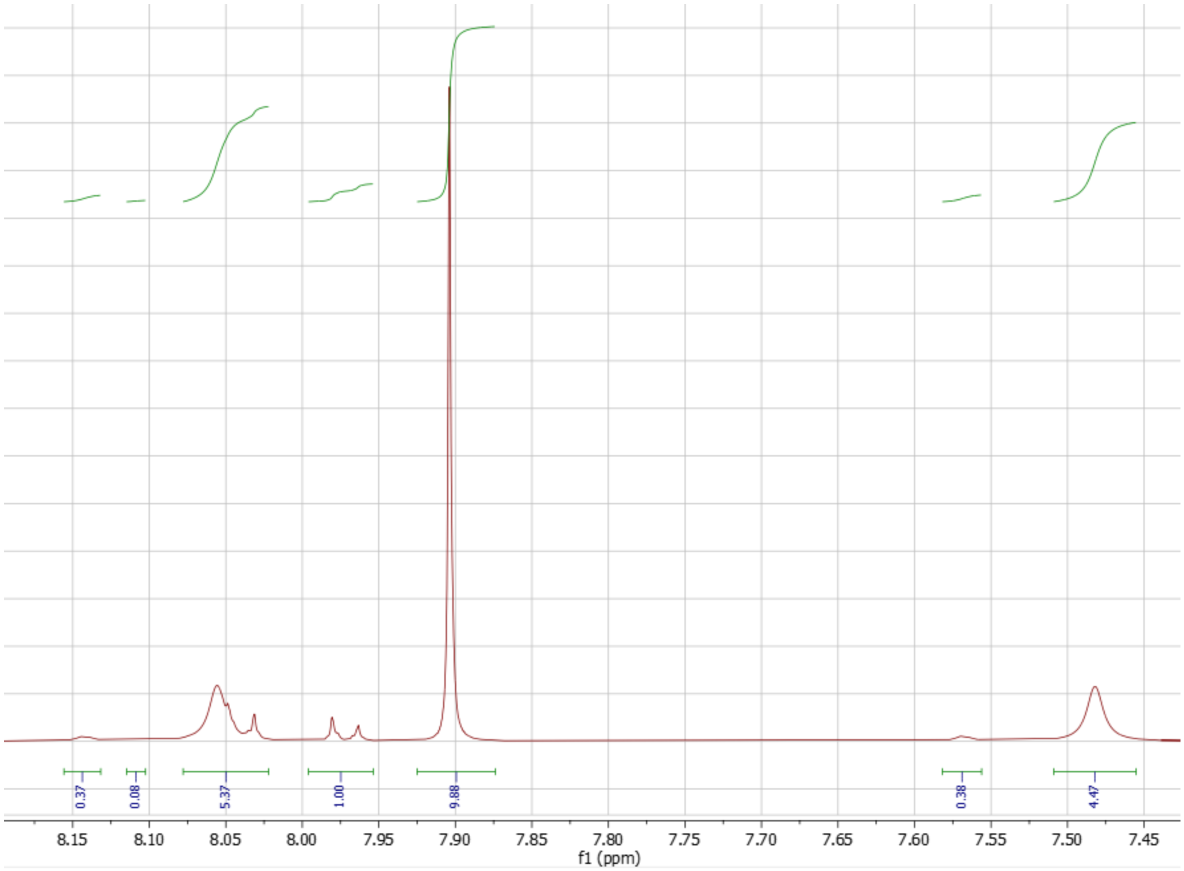

Figure S5. 1H NMR the between 7.45 and 8.15PPM of the of the recovered solid after a 45 min run at 50°C. The spectrum is zoomed into the aromatic region to illustrate the analysis. Terephthalamide 1H NMR ((500MHz, DMSO) δ 8.06 (s, 2H), 7.91 (s, 4H), 7.4

The integrals of the NMR peaks (or areas under the curve) in the aromatic region (approximately 6.5PPM to 8.5PPM) are used to determine how much of each solid species is present (see Figure S5). The aromatic hydrogens are an excellent metric to use to compare the relative amounts of each of the compounds because each compound has the same number (4) of aromatic protons. The aromatic peak locations are discussed in the results session of this paper. Since there is an overlap between the one of the aromatic 2-hydroxyethyl 4-carbamoylbenzoate (HCB) peaks and an amide of terephthalamide (TPD), the total integral for the aromatic protons on HCB was taken as double the integral value of the peak without overlap. Letting f(x) be the NMR curve, $I_{solid,BHET}$, $I_{solid,HCB}$, and $I_{solid,TPD}$ are defined in Eqns. S1-S3

$$I_{solid,BHET} = \int_{8.10PPM}^{8.14PPM} f(x)dx \tag{S1}$$

$$I_{solid,HCB} = 2\int_{8.02PPM}^{8.07PPM} f(x)dx \tag{S2}$$

$$I_{solid,TPD} = \int_{7.89PPM}^{7.93PPM} f(x)dx \tag{S3}$$

The molar ratios of each of the solids are calculated using Eqns. S4-S5.

$$R_{solid,BHET} = \frac{I_{solid,BHET}}{I_{solid,BHET} + I_{solid,HCB} + I_{solid,TPD}} \tag{S4}$$

$$R_{solid,HCB} = \frac{I_{solid,HCB}}{I_{solid,BHET} + I_{solid,HCB} + I_{solid,TPD}} \tag{S5}$$

$$R_{solid,TPD} = \frac{I_{solid,TPD}}{I_{solid,BHET} + I_{solid,HCB} + I_{solid,TPD}} \tag{S6}$$

where $R_{solid,BHET}$, $R_{solid,HCB}$, and $R_{solid,TPD}$ are the molar ratios of bis(2-hydroxyethyl) terephthalate, 2-hydroxyethyl 4-carbamoylbenzoate, and terephthalamide, respectively as determined by the integrals in the aromatic region of the NMR. The overall molecular weight of the solid sample is calculated using Eqn. S7.

$$Mw_{solid,overall} = R_{solid,BHET} Mw_{BHET} + R_{solid,HCB} Mw_{HCB} + R_{solid,TPD} Mw_{TPD} \tag{S7}$$

In Eqn. S7, Mw_{BHET}, Mw_{HCB}, and Mw_{TPD}, are the molecular weights of BHET, HCB and TPD, respectively. The mass recovered, $m_{recoverd}$ is divided by the overall molecular weight of the sample to determine the number of moles in the solid in Eqn. S8.

$$n_{solid} = \frac{m_{recoverd}}{Mw_{solid,overall}} \tag{S8}$$

The initial moles of the solid reactant (BHET) are determined by Eqn. S9.

$$n_{initial,BHET} = \frac{m_{initial,BHET}}{M_{w,BHET}} \tag{S9}$$

Using an overall mole balance, it is assumed that any non-recovered sample remained dissolved in the ethylene glycol solution in Eqn. S10.

$$n_{dissolved} = n_{initial,BHET} - n_{solid} \tag{S10}$$

One challenge is the solubility of the product in the ethylene glycol solution. Much of the BHET and HCB remains dissolved after the reaction is thermally quenched. A proton NMR (Figure S2) was taken of the dissolved sample to quantify the molar ratio of each component. The ratios were determined in the same way however the peaks were slightly shifted due to the presence of ethylene glycol and ammonia. It is also noted that the amide peaks disappeared, likely from an interaction with the ammonia present in the solvent. The molar ratios of each of the dissolved species were calculated in Eqns. S11-S12.

$$R_{dissolved,BHET} = \frac{I_{dissolved,BHET}}{I_{dissolved,BHET} + I_{dissolved,HCB} + I_{dissolved,TPD}} \tag{S11}$$

$$R_{dissolved,HCB} = \frac{I_{dissolved,HCB}}{I_{dissolved,BHET} + I_{dissolved,HCB} + I_{dissolved,TPD}} \tag{S12}$$

$$R_{dissolved,TPD} = \frac{I_{dissolved,TPD}}{I_{dissolved,BHET} + I_{dissolved,HCB} + I_{dissolved,TPD}} \quad \text{(S13)}$$

A solid fraction and dissolved fraction were calculated in Eqns. S14-S15

$$F_{solid} = \frac{n_{solid}}{n_{initial,BHET}} \quad \text{(S14)}$$

$$F_{dissolved} = \frac{n_{dissolved}}{n_{initial,BHET}} \quad \text{(S15)}$$

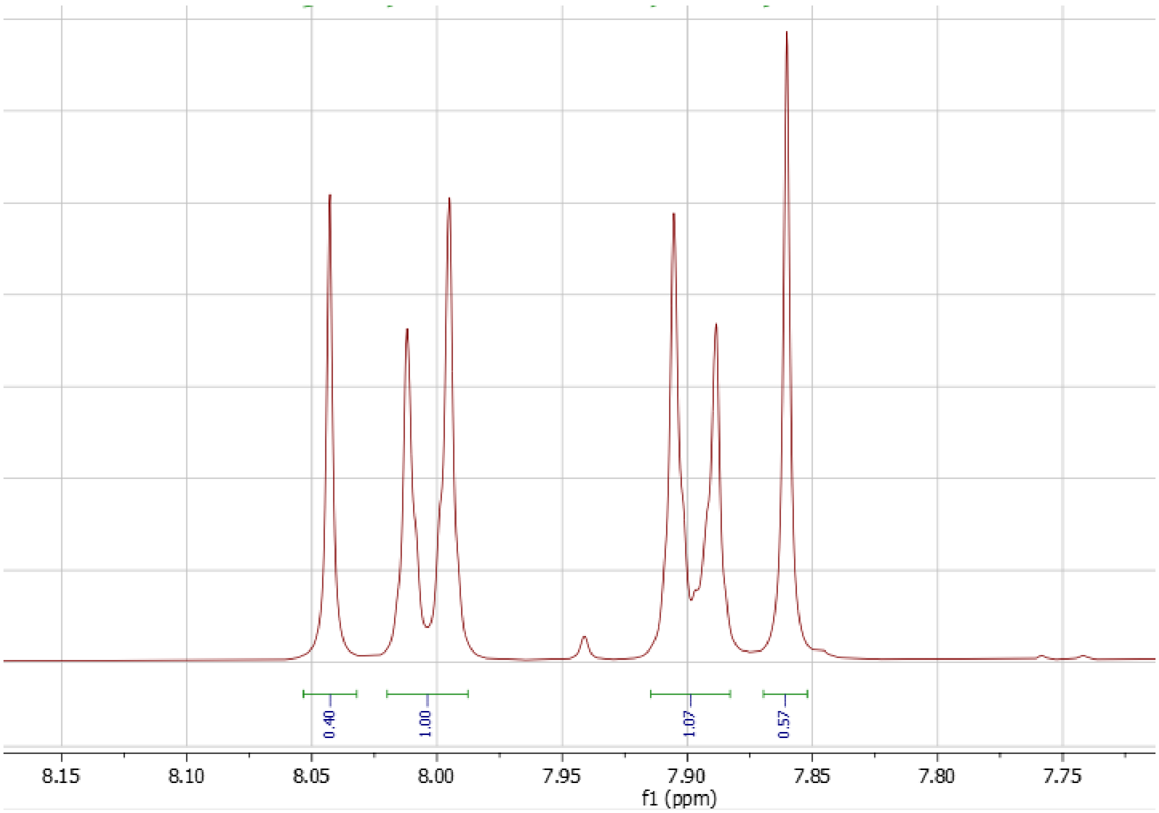

Figure S6. Proton NMR of the sample that remained dissolved in the ethylene glycol and ammonia solution after a reaction at 50 °C for 45 min. The peaks are shifted from their locations using just DMSO as the solvent. The aromatic peaks are BHET (8.04(s, 4H)), HCB (8.02-7.99(m, 2H), 7.92-7.88(m, 2H), TPD (7.86(s, 4H)). These peak locations are not perfectly consistent across all samples but they only change ±.05PPM and their order stays consistent.

With Eqns. S14-S15, the overall molar ratios of the aromatic components can be calculated using Eqns. S16-S18.

$$R_{overall,BHET} = F_{solid} R_{solid,BHET} + F_{dissolved} R_{dissolved,BHET} \quad \text{(S16)}$$

$$R_{overall,HCB} = F_{solid} R_{solid,HCB} + F_{dissolved} R_{dissolved,HCB} \quad \text{(S17)}$$

$$R_{overall,TPD} = F_{solid} R_{solid,TPD} + F_{dissolved} R_{dissolved,TPD} \quad \text{(S18)}$$

The ammonia solution, present in the reaction, needs to be accounted for to determine the mole fractions. The known molarity of the solution is converted into a mole fraction of the solute in solution using Eqn. S19.

$$M_{w,solute} * C * V = m_{solute} \quad \text{(S19)}$$

In Eqn. S19, $M_{w,solute}$ is the molecular weight of the compound in g/mol, C is the concentration, V the volume of the solution used, and m is the mass of the compound in that solution. The mass of the solvent is given in Eqn. S20,

$$m_{solvent} = \rho * V - m_{solute} \quad \text{(S20)}$$

where ρ is the density of the solution. The mass of the solvent and solute were converted to moles by Eqns. S21-S22.

$$n_{solvent} = \frac{m_{solvent}}{M_{w,solvent}} \quad \text{(S21)}$$

$$n_{solute} = \frac{m_{solute}}{M_{w,solute}} \quad \text{(S22)}$$

Here $M_{w,solvent}$ is the molecular weight of the solvent and $M_{w,solute}$ is the molecular weight of the solute. In this study the solvent is methanol and the solute is ammonia as shown in Eqns. S24-S25.

$$n_{solvent} = n_{ethylene\ glycol} \quad \text{(S24)}$$

$$n_{solute} = n_{ammonia} \quad \text{(S25)}$$

The mole fraction of ammonia ($x_{ammonia}$), ethylene glycol ($x_{ethylene\,glycol}$), and the aromatics ($x_{aromatics}$) were determined by dividing the initial moles of ammonia by the sum of the moles in the reactor using Eqns. S26-S28.

$$x_{ammonia} = \frac{n_{ammonia}}{n_{ammonia} + n_{ethylene\ glycol} + n_{initial,BHET}} \quad \text{(S26)}$$

$$x_{ethylene\ glycol} = \frac{n_{ethylene\ glycol}}{n_{ammonia} + n_{ethylene\,glycol} + n_{initial,BHET}} \quad \text{(S27)}$$

$$x_{aromatics} = \frac{n_{initial,BHET}}{n_{ammonia} + n_{ethylene\ glycol} + n_{initial,BHET}} \quad \text{(S28)}$$

It is assumed that the mole fraction of ammonia is constant because it is in large excess (8 to 32 times the stoichiometric amount). The mole fraction of ethylene glycol is also assumed to be constant because of its large molar excess. The molar ratios of the solids, as determined by the NMR spectroscopy, are multiplied by the solid mole fraction to obtain their individual mole fractions at each time step using Eqns. S29-S31.

$$x_{BHET} = x_{aromatics} * R_{dry,DMT} \quad \text{(S29)}$$

$$x_{HCB} = x_{aromatics} * R_{dry,MCB} \quad \text{(S30)}$$

$$x_{TPD} = x_{aromatics} * R_{dry,TPD} \tag{S31}$$

2.1 Example of determining mole fractions

An example of determining the mole fractions using Eqns. S1-S31 and the values in Figure S5 follows.

$$I_{solid,BHET} = \int_{8.10PPM}^{8.14PPM} f(x)dx = .08 \tag{S32}$$

$$I_{solid,HCB} = 2\int_{8.02PPM}^{8.07PPM} f(x)dx = 2.00 \tag{S33}$$

$$I_{solid,TPD} = \int_{7.89PPM}^{7.93PPM} f(x)dx \tag{S34}$$

The integrals were added together to get the total moles of aromatic hydrogens. The integrals for each aromatic peak were divided by the total moles of aromatic hydrogens to get a dry mole fraction in Eqns. S35-S36.

$$R_{solid,BHET} = \frac{0.08}{0.08 + 2.00 + 9.88} = .007 \tag{S35}$$

$$R_{solid,HCB} = \frac{2.00}{0.08 + 2.00 + 9.88} = .167 \tag{S36}$$

$$R_{solid,TPD} = \frac{9.88}{0.08 + 2.00 + 9.88} = .826 \tag{S37}$$

The calculation of the overall molecular weight is shown in Eqns. S38-S39.

$$Mw_{solid,overall} = .007 * 254.24\frac{g}{mol} + .167 * 209.20\frac{g}{mol} + .826 * 164.16\frac{g}{mol} \tag{S38}$$

$$Mw_{solid,overall} = 172.29\frac{g}{mol} \tag{S39}$$

The mass recovered was divided by the overall molecular weight of the sample in Eqn. S40. In this case, the mass recovered as a solid was 0.0006 g.

$$n_{solid} = \frac{0.0006\ \mathrm{g}}{172.29\ g/mol} = 3.48E - 6\ mol \tag{S40}$$

The initial moles of the solid reactant (BHET) were determined as shown in Eqn. S41.

$$n_{initial,BHET} = \frac{1.00\ g}{254.24\ g/mol} = .00393\ mol \tag{S41}$$

Using an overall mole balance, it was assumed that any non-recovered sample remained dissolved in the ethylene glycol solution. The number of moles that remained dissolved are calculated in Eqn. S42.

$$n_{dissolved} = .00393\ mol - 3.48E - 6\ mol = .00393\ mol \tag{S42}$$

The fraction dissolved and recovered as a solid are calculated in Eqns. S43-S44.

$$F_{solid} = \frac{3.48E - 6\, mol}{.00393\, mol} = 0 \quad \text{(S43)}$$

$$F_{dissolved} = \frac{.00393\, mol}{.00393\, mol} = 1 \quad \text{(S44)}$$

It is noted that the significant numbers of this example result in 100% of the sample remaining dissolved in the ethylene glycol. This is not always the case.

To determine the composition of the dissolved fraction, the integrals of peaks in the proton NMR of the dissolved sample (Figure S2) are determined and used to calculate the molar ratios of each component as shown in Eqns. S45-S47.

$$I_{dissolved,BHET} = \int_{8.02ppm}^{8.06ppm} f(x)\,dx = .40 \quad \text{(S45)}$$

$$I_{dissolved,HCB} = \int_{7.99}^{8.02} f(x)\,dx + \int_{7.88}^{7.92} f(x)\,dx = 2.07 \quad \text{(S46)}$$

$$I_{dissolved,TPD} = \int_{7.84}^{7.87} f(x)\,dx = .57 \quad \text{(S47)}$$

The molar ratios for each species in the dissolved sample are calculated in Eqns. S48-S50.

$$R_{dissolved,BHET} = \frac{0.40}{0.40 + 2.07 + .57} = .132 \quad \text{(S48)}$$

$$R_{dissolved,MCB} = \frac{2.00}{0.40 + 2.07 + .57} = .681 \quad \text{(S49)}$$

$$R_{dissolved,TPD} = \frac{9.88}{0.40 + 2.07 + .57} = .188 \quad \text{(S50)}$$

With these fractions the overall molar ratios of the aromatic components can be calculated.

$$R_{overall,BHET} = 0 * .007 + 1 * .132 = .132 \quad \text{(S51)}$$

$$R_{overall,HCB} = 0 * .167 + 1 * .681 = .681 \quad \text{(S52)}$$

$$R_{overall,TPD} = 0 * .826 + 1 * .188 = .188 \quad \text{(S53)}$$

The known molarity of the solution was multiplied by the molar mass of ammonia to get the number of grams of ammonia in a mL of solution. At other temperatures the solution was diluted to slow the reaction. The concentration used to determine the mole fraction should follow Table 2 in the published document.

$$\frac{17.03g}{mol} * 10.9\, mol/L = .186\, g/mL \quad \text{(S54)}$$

A 1.0 mL basis was used to determine the number of moles of methanol in a mL of the solution as shown in Eqns. S55-S57. The solution density was found to be 1.00 g/mL. In 1 mL there is 1.00 g of solution and .186 g of ammonia.

$$m_{solvent} = m_{ethylene\ glycol} = 1.00g - .186g = .814\ g \tag{S55}$$

$$n_{ethylene\,glycol} = \frac{.660g}{62.07\ g/mol} = 13.1\ mmol \tag{S56}$$

$$n_{ammonia} = \frac{.186g}{17.04\ g/mol} = 10.9\ mmol \tag{S57}$$

The mmols of ammonia and methanol present in the reactor were determined by multiplying the molarity by 10 mL in Eqns. S58-S60.

$$n_{methanol} = 13.1\ mmol/mL * 10mL = 131\ mmol \tag{S58}$$

$$n_{ammonia} = 7\ mol/L * 15\ mL = 109\ mmol \tag{S59}$$

$$n_{initial,DMT} = \frac{1.00g}{254.24g/mol} = 3.93mmol \tag{S60}$$

The mole fractions of ammonia, ethylene glycol, and aromatics are calculated in Eqns. S61-S62.

$$x_{ammonia} = \frac{109mmol}{109mmol + 131mmol + 3.93mmol} = .447 \tag{S61}$$

$$x_{ethylene\ glycol} = \frac{131mmol}{109mmol + 131mmol + 3.93mmol} = .537 \tag{S62}$$

$$x_{aromatics} = \frac{3.93mmol}{109mmol + 131mmol + 3.93mmol} = .0161 \tag{S63}$$

The overall molar ratios (Eqns. 51-53) was multiplied with the mole fraction of the aromatics in Eqns. S64-S66.

$$x_{BHET,45min,50^0C} = .0161 * .132 = .00212 \tag{S64}$$

$$x_{HCB,45min,50^0C} = .0161 * .681 = .00110 \tag{S65}$$

$$x_{TPD,45min,50^0C} = .0161 * .188 = .00302 \tag{S66}$$

3.0 Gibbs free energy of reaction

Thermodynamic equilibrium for the reactions are approximated by estimating the Gibbs free energy of reaction. This estimation requires the Gibbs free energy of formation of each compound. The NIST Webbook (https://webbook.nist.gov/chemistry/) provides Gibbs free energy of formation values for ammonia and ethylene glycol. The reported Gibbs free energy of formation for Ammonia (gas) and ethylene glycol (liquid) are -16.4 kJ/mol and -338.9 kJ/mol respectively.

Since the value of the Gibbs energy of formation is given for ammonia as a gas and ammonia is dissolved in methanol, the value must be converted to ammonia as a liquid; this is done using Eqn. S67.

$$\Delta G_f^{liq}(298, 1bar) = \Delta G_f^{gas} + V_L\left(1 - P_{298}^{SAT}\right) + RTln\left(\frac{P_{298}^{SAT}}{1bar}\right) \quad \text{(S67)}$$

Where ΔG_f^{liq} is the Gibbs free energy of formation in the liquid phase, ΔG_f^{gas} is the Gibbs free energy of formation in the gas phase, V_L is molar volume, P_{298}^{SAT} is the saturation pressure at 298K, R is the ideal gas constant, and T is absolute temperature. The saturation pressure at 298K is shown in Eqn. S68.

$$P_{298}^{SAT} = 10bar \quad \text{(S68)}$$

The density of liquid ammonia is expressed in Eqn. S35.

$$\rho = 0.73\frac{g}{cm^3}\left(10^6\frac{cm^3}{m^3}\right)\left(\frac{1kg}{10^3 g}\right) = 730\ kg/m^3 \quad \text{(S69)}$$

The molar volume of ammonia is calculated by dividing the molecular weight by the density of ammonia in Eqn. S70.

$$V_L = \frac{1}{730\frac{kg}{m^3}}17\frac{g}{mol}\left(\frac{1kg}{10^3 g}\right) = 2.33 * 10^{-5}m^3/mol \quad \text{(S70)}$$

The volume portion of Eqn. S33 was calculated in Eqn. S71.

$$V_L\left(1 - P_{298}^{SAT}\right) = 2.33 * 10^{-5}\frac{m^3}{mol}\left(1 * 10^5\frac{N}{m^2} - 10 * 10^5\frac{N}{m^2}\right) = -2.1\frac{Nm}{mol} = 2 * 10^{-3}kJ/mol \quad \text{(S71)}$$

The pressure portion of Eqn. S33 is then used in Eqn. S72.

$$RTln\left(\frac{P_{298}^{SAT}}{1bar}\right) = 2.48\frac{kJ}{mol} * ln\left(\frac{10bar}{1bar}\right) = 5.71\ kJ/mol \quad \text{(S72)}$$

Eqns. S37-S38 are substituted into Eqn. S33 resulting in Eqn. S73.

$$\Delta G_{f,ammonia}^{liq}(298, 1bar) = -16.4\frac{kJ}{mol} + .002\frac{kJ}{mol} + 5.71\frac{kJ}{mol} = \mathbf{-10.69\ kJ/mol} \quad \text{(S73)}$$

To determine Gibbs free energy of formations for species not in the NIST Webbook, the group contribution developed by L. Constantinou was employed [1]. This method uses Eqn. S74.

$$\Delta G_f = g_0 + \sum N_j g_{1j} + \sum M_j g_{2j} \quad \text{(S74)}$$

Where g_0 is a constant, g_{1j} is the Gibbs free energy of formation of primary functional group *j*, N_j is the number of occurrences of primary functional group *j*, g_{2j} is the Gibbs free energy of formation of primary functional group *j*, and M_j is the number of occurrences of primary functional group *j*.

Table S1. The group contribution calculation of Gibbs energy of formation for BHET

Bis(2-hydroxyethyl) terephthalate (BHET)	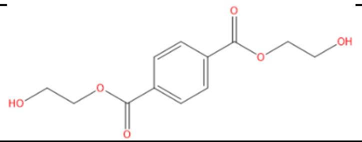		
Primary Group	# of occurrences	g_{ij} (kJ/mol)	Contribution (kJ/mol)
CH_2	4	8.231	32.924
OH	2	-158.589	-317.178
COO	2	-281.495	-562.990
AC	2	30.485	60.970
ACH	4	22.533	90.132
Secondary group			
ACOO	2	-7.415	-14.830
Constant			
g_0	1	-14.828	-14.828
ΔG_f^{DMT}	-	-	**-725.80**

Table S2. The group contribution calculation of Gibbs energy of formation for HCB

(2-hydroxyethyl) 4-carbamoylbenzoate (HCB)	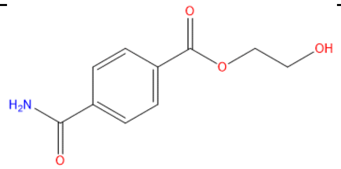		
Primary Group	# of occurrences	g_{ij} (kJ/mol)	Contribution (kJ/mol)
CH_2	2	8.231	16.462
OH	1	-158.589	-158.589
COO	1	-281.495	-281.495
$CONH_2$	1	-136.742	-136.742
AC	2	30.485	60.970
ACH	4	22.533	90.132
Secondary group			
ACOO	1	-7.415	-7.415
Constant			
g_0	1	-14.828	-14.828
ΔG_f^{DMT}	-	-	**-431.51**

Table S3. The group contribution calculation of Gibbs energy of formation for TPD

Terephthalamide (TPD)	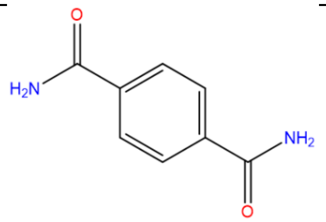		
Primary Group	# of occurrences	g_{ij} (kJ/mol)	Contribution (kJ/mol)
$CONH_2$	2	-136.742	-136.742
AC	2	30.485	60.970
ACH	4	22.533	90.132
Constant			
g_0	1	-14.828	-14.828
ΔG_f^{DMT}	-	-	**-137.21**

3.1 First reaction step

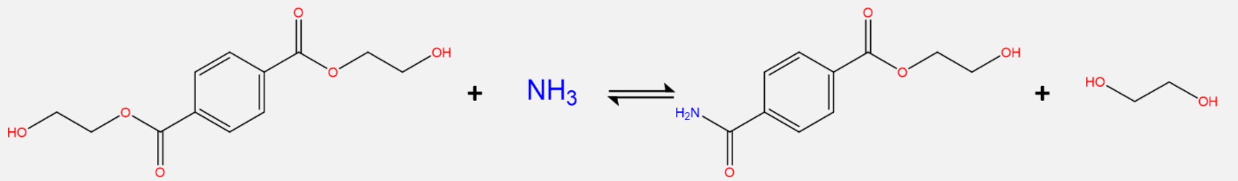

Bis(2-hydroxyethyl) terephthalate (BHET) + Ammonia ⇌ (2-hydroxyethyl) 4-carbamoylbenzoate (HCB) + Ethylene glycol

Scheme S1. The first reaction step expressed as an equilibrium reaction

The Gibbs free energy of reaction can be determined using Eqn. S75.

$$\Delta G_{rxn} = \sum \nu_i \Delta G_f^i = \left(-431.51\frac{kJ}{mol} - 338.9\frac{kJ}{mol}\right) - \left(-725.80\frac{kJ}{mol} - 10.69\frac{kJ}{mol}\right) \quad \text{(S75)}$$

Where ν_i is the stoichiometric coefficient for species i and ΔG_f^i is the Gibbs energy of formation for species i. The resulting Gibbs free energy of reaction for the first reaction step is found in Eqn. S 76.

$$\boldsymbol{\Delta G_{rxn1} = -33.9\ kJ/mol} \quad \text{(S76)}$$

The Gibbs energy of reaction can be related to the equilibrium constant by Eqns. S77-S78.

$$\Delta G_{rxn} = -RT lnK \quad \text{(S77)}$$

$$K = \exp\left(-\frac{\Delta G_{rxn}}{RT}\right) \quad \text{(S78)}$$

At T=25°C the equilibrium constant was found in Eqns. S79-S80.

$$K = \exp\left(\frac{33.9\frac{kJ}{mol}}{0.008314\frac{kJ}{molK} * 298.15K}\right) \tag{S79}$$

$$K = 875{,}000 \tag{S80}$$

At T=100°C the equilibrium constant was found in Eqns. S81-S82.

$$K = \exp\left(\frac{33.9\frac{kJ}{mol}}{0.008314\frac{kJ}{molK} * 373.15K}\right) \tag{S81}$$

$$K = 55{,}900 \tag{S82}$$

3.2 Second reaction step

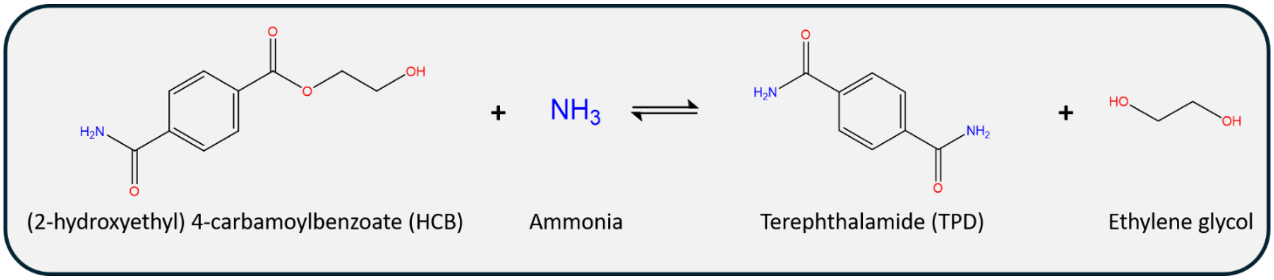

Scheme S2. The second reaction step expressed as an equilibrium reaction

The Gibbs free energy of the second reaction step can be determined with Eqn. S83. The result is the same value because both reactions are the same in terms of functional groups.

$$\Delta G_{rxn} = \sum \nu_i \Delta G_f^i = \left(-137.21\frac{kJ}{mol} - 338.9\frac{kJ}{mol}\right) - \left(-431.51\frac{kJ}{mol} - 10.69\frac{kJ}{mol}\right) \tag{S83}$$

$$\Delta G_{rxn2} = -33.9\ kJ/mol \tag{S84}$$

The Gibbs free energy of reaction can be related to the equilibrium constant by Eqns. S77-S78. At T=25°C the equilibrium constant was found in Eqns. S87-S88.

$$K = \exp\left(\frac{33.9\frac{kJ}{mol}}{0.008314\frac{kJ}{molK} * 298.15K}\right) \tag{S87}$$

$$K = 875000 \tag{S88}$$

At T=100°C the equilibrium constant was found in Eqns. S89-S90.

$$K = \exp\left(\frac{33.9\frac{kJ}{mol}}{0.008314\frac{kJ}{molK} * 373.15K}\right) \tag{S89}$$

$$K = 55900 \tag{S90}$$

3.3 Overall reaction

To determine the Gibbs free energy of the overall reaction, the two Gibbs free energies are summed. The product is taken of the two equilibrium constants to determine the overall equilibrium constant resulting in Eqns. S91-S93.

$$\Delta G_{rxn} = -67.8 kJ/mol \quad \text{(S91)}$$

$$K_{overall,25^0C} = 875000^2 = 7.7E11 \quad \text{(S92)}$$

$$K_{overall,100^0C} = 55900^2 = 3.1E9 \quad \text{(S93)}$$

These large values are consistent with experiments where quantitative yields are obtained.

3.4 Calculation of Equilibrium Conversion

Let y be the moles of DMT reacted (i.e. conversion)

Table S4. The number of moles of each compound at time=0 and at equilibrium for the reaction at a setpoint temperature of T=100C

Compound	At time=0 (mol)	At Equilibrium (mol)
Bis(2-hydroxyethyl) terephthalate (BHET)	1	$N_{DMT} = 1 - y$
Terephthalamide (TPD)	0	$N_{TPD} = y$
Ammonia	7	$N_{Ammonia} = 7 - 2y$
Ethylene Glycol	55	$N_{methanol} = 55 + 2y$
Total	63	63

The equilibrium can be expressed as a function of the activities of the products and reactants in Eqn. S94.

$$K_{overall} = \frac{a_{TPD} a_{methanol}^2}{a_{DMT} a_{ammonia}^2} \quad \text{(S94)}$$

Assuming an Ideal solution (the activity is assumed to be equal to the mole fraction) as seen in Eqn. S95.

$$K_{overall} = \frac{x_{TPD} x_{methanol}^2}{x_{DMT} x_{ammonia}^2} \quad \text{(S95)}$$

Substituting the mole fractions from Table S4 into Eqn. S95 results in Eqn. 96.

$$K_{overall} = \frac{\frac{y}{63}\left(\frac{55+2y}{63}\right)^2}{\frac{1-y}{63}\left(\frac{7-2y}{63}\right)^2} \quad \text{(S96)}$$

Simplifying and rearranging result in Eqns. S97-S99.

$$K_{overall} = \frac{y(55+2y)^2}{(1-y)(7-2y)^2} \quad \text{(S97)}$$

$$K_{overall}(1-y)(7-2y)^2 = y(55+2y)^2 \qquad \text{(S98)}$$

$$K_{overall}(1-y)(7-2y)^2 - y(55+2y)^2 = 0 \qquad \text{(S99)}$$

The $K_{overall,100^oC} = 3.13E9$ value can be placed in for K_o resulting in Eqn. 100.

$$3.13E9(1-y)(7-2y)^2 - y(55+2y)^2 = 0 \qquad \text{(S100)}$$

Now that the equation is in residual form, the equation can be solved numerically (Excel was used).

$$y^{equil} = 0.998 \qquad \text{(S101)}$$

As indicated by the large values of the equilibrium constants a very high conversion is predicted.

4.0 Solution to the coupled ordinary differential equations

The differential equations for the mole fraction model fit were solved using Laplace transforms in the following way.

Table of chemicals and their name and letter and reaction scheme

**Chemical Structure**	**Name**	**Species subscript**
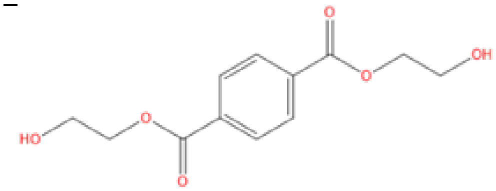	Bis(2-hydroxyethyl) terephthalate (BHET)	A
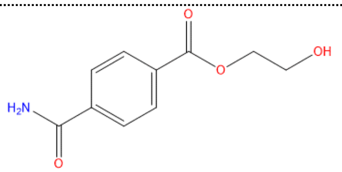	(2-hydroxyethyl) 4-carbamoylbenzoate (HCB)	B
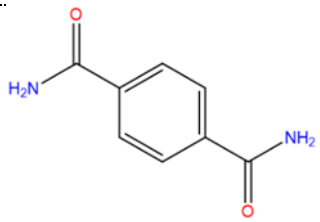	Terephthalamide	C
NH_3	Ammonia	F
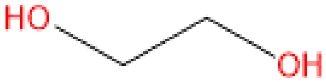	Ethylene glycol	G

The differential equations for the BHET kinetics comprise Eqns. S102-S104

$$\frac{dA}{dt} = -k_1 AFG \tag{S102}$$

$$\frac{dB}{dt} = k_1 AFG - k_2 BFG \tag{S103}$$

$$\frac{dC}{dt} = k_2 BFG \tag{S104}$$

Where k_1 and k_2 are the rate constants for the first and second reaction steps respectively and A, B, C, F and G are the mole fraction of BHET, HCB, TPD, ammonia, and ethylene glycol respectively. The concentration of ammonia and ethylene glycol (G) are assumed to be constant in Eqns. S105-S106.

$$\frac{dF}{dt} = 0 \tag{S105}$$

$$\frac{dG}{dt} = 0 \tag{S106}$$

The equations are solved in Laplace space, the transform of Eqn. S102 is

$$sA(s) - A_0 = -k_1 A(s) FG \tag{S107}$$

$$A(s) = \frac{A_0}{s + k_1 FG} \tag{S108}$$

where s is the Laplace space variable and A_0 is the initial mole fraction of DMT. The final expression is shown in Eqn. S109.

$$A(t) = A_0 \exp(-k_1 FGt) \tag{S109}$$

Eqn. S69 was transformed into the Laplace domain to give S110

$$sB(s) - B_0 = k_1 A(s) FG - k_2 B(s) FG \tag{S110}$$

The appropriate initial condition is given by S111,

$$B_0 = 0 \tag{S111}$$

yielding Eqn. S112.

$$sB(s) = k_1 \frac{A_0}{s + k_1 FG} FG - k_2 B(s) FG \tag{S112}$$

Rearrangement gives,

$$B(s) = \frac{\left(\frac{k_1 A_0 FG}{s + k_1 FG}\right)}{s + k_2 FG} \tag{S113}$$

$$B(s) = \frac{k_1 A_0 FG}{(s + k_1 FG)(s + k_2 FG)} \tag{S114}$$

Partial fraction expansion of S114 gives S115

$$B(s) = \frac{X}{s} + \frac{Y}{s + k_1 FG} + \frac{Z}{s + k_2 FG} \tag{S115}$$

where X, Y, and Z are the numerators to be solved for in the partial fraction expansion. For simplification, a_1 and a_2 are defined in Eqns. S116-S117.

$$a_1 = k_1 FG \tag{S116}$$

$$a_2 = k_2 FG \tag{S117}$$

Substituting a_1 and a_2 and setting X to 0 gives Eqn. S118.

$$\frac{k_1 A_0 FG}{(s + k_1 FG)(s + k_2 FG)} = \frac{Y}{s + a_1} + \frac{Z}{s + a_2} \tag{S118}$$

Determining the common denominator, and multiplying it to both sides of Eqn. S118 gives Eqn. S119.

$$k_1 A_0 FG = Y(s + a_2) + Z(s + a_1) \tag{S119}$$

Distributing Y and Z and factoring s, results in Eqn. S120.

$$s * 0 + k_1 A_0 FG = s(Y + Z) + (Ya_2 + Za_1) \tag{S120}$$

Separating into an s term and a constant term yields Eqns. S121-S122

s term	$Y + Z = 0$	(S121)
Constant term	$Ya_2 + Za_1 = k_1 A_0 FG$	(S122)

Solving for Z in S121 gives S123.

$$Z = -Y \tag{S123}$$

Plugging Eqn. S123 into Eqn. S122, results in Eqn. S124.

$$k_1 A_0 FG = Ya_2 + (-Y)a_1 \tag{S124}$$

Algebraically solving for Y gives Eqn. S125.

$$\frac{(k_1 A_0 FG)}{a_2 - a_1} = Y \tag{S125}$$

Plugging S116-S117 into S125 results in S126.

$$Y = \frac{k_1 A_0 FG}{k_2 FG - k_1 FG} \tag{S126}$$

Substituting Eqn. S126 into Eqn. S123 gives Eqn. S127.

$$Z = -\frac{k_1 A_0 FG}{k_2 FG - k_1 FG} \tag{S127}$$

The inverse Laplace transform of S115 is S128.

$$B(t) = X + Yexp(-k_1 FGt) + Zexp(-k_2 FGt) \tag{S128}$$

Plugging the values obtained for X, Y, and Z into S94 and simplifying yields the solution in Eqn. S 129.

$$B(t) = \frac{k_1 A_0}{k_2 - k_1} exp(-k_1 FGt) - \frac{k_1 A_0}{k_2 - k_1} exp(-k_2 FGt) \tag{S129}$$

Eqn. S104 was transformed into the Laplace domain and C(s) was solved for in Eqns. S 130,

$$sC(s) - C_0 = k_2 B(s) FG \tag{S130}$$

where C_0 is the initial mole fraction of TPD. An appropriate initial mole fraction of 0 was chosen,

$$C_0 = 0 \tag{S131}$$

yielding Eqn. S132.

$$C(s) = \frac{k_2 B(s) FG}{s} \tag{S132}$$

Plugging Eqn. S80 into Eqn. 98 and subsequent rearrangement results in Eqns. S 133-S134.

$$C(s) = \frac{k_2 \left(\frac{k_1 A_0 FG}{(s + k_1 FG)(s + k_2 FG)} \right) FG}{s} \tag{S133}$$

$$C(s) = \frac{k_1 k_2 A_0 F^2 G^2}{s(s + k_1 FG)(s + k_2 FG)} \tag{S134}$$

Partial fraction expansion of S134 gives S135.

$$\frac{k_1 k_2 A_0 F^2 G^2}{s(s + k_1 FG)(s + k_2 FG)} = C(s) = \frac{X}{s} + \frac{Y}{s + k_1 FG} + \frac{Z}{s + k_2 FG} \tag{S135}$$

For simplification, a_1 and a_2 are defined in Eqns. S136-S137.

$$a_1 = k_1 FG \tag{S136}$$

$$a_2 = k_2 FG \tag{S137}$$

Determining the common denominator, and multiplying it to both sides of Eqn. S1135 gives Eqn. S138.

$$k_1 k_2 A_0 F^2 G^2 = X(s + a_1)(s + a_2) + Y(s + a_2)s + Z(s + a_1)s \tag{S138}$$

Distributing results in S139.

$$k_1 k_2 A_0 F^2 G^2 = Xs^2 + Xsa_1 + Xsa_2 + Xa_1 a_2 + Ys^2 + Ysa_2 + Zs^2 + Zsa_1 \tag{S139}$$

Separating S139 into s^2, s, and constant terms yields Eqns. S140-S141.

s^2 term $$X + Y + Z = 0 \tag{S140}$$

s term $$X(a_1 + a_2) + Ya_2 + Za_1 = 0 \tag{S141}$$

Constant term $$k_1 k_2 A_0 F^2 G^2 = X a_1 a_2 \tag{S142}$$

Solving for X in Eqn. S142 and substituting Eqns. S136-S137 results in Eqn. S143.

$$X = \frac{k_1 k_2 A_0 F^2 G^2}{a_1 a_2} = \frac{k_1 k_2 A_0 F^2 G^2}{k_1 FG(k_2 FG)} = A_0 \tag{S143}$$

Substituting S143 into S140 yields S144.

$$A_0 + Y + Z = 0 \tag{S144}$$

Solving for Z results in Eqn. S145.

$$Z = -A_0 - Y \tag{S145}$$

Eqns. S145 and S143 are substituted into Eqn. S141 to yield Eqn. S146.

$$0 = A_0(a_1 + a_2) + Ya_2 + (-A_0 - Y)a_1 \tag{S146}$$

Rearrangement results in Eqns. S147-S148.

$$-A_0(a_1 + a_2) + A_0 a_1 = Ya_2 - Ya_1 \tag{S147}$$

$$\frac{(-A_0 a_2)}{a_2 - a_1} = Y \tag{S148}$$

Substituting Eqns. S136-S137 into S148 gives Eqn. S149.

$$Y = \frac{-k_2 A_0}{k_2 - k_1} \tag{S149}$$

Substituting Eqn. S149 into Eqn. S145 yields Eqn. S150.

$$Z = \frac{k_2 A_0}{k_2 - k_1} - A_0 \tag{S150}$$

Rearrangement of Eqn. S150 shown in Eqns. S151-S152 results in Eqn. S153.

$$Z = A_0\left(\frac{k_2}{k_2 - k_1} - 1\right) \tag{S151}$$

$$Z = A_0\left(\frac{k_2}{k_2 - k_1} - \frac{k_2 - k_1}{k_2 - k_1}\right) \tag{S152}$$

$$Z = A_0\left(\frac{k_1}{k_2 - k_1}\right) \tag{S153}$$

The inverse Laplace transform of Eqn. S135 results in Eqn. S154.

$$C(t) = X + Yexp(-k_1FGt) + Zexp(-k_2FGt) \quad \text{(S154)}$$

Substituting Eqns. S143, S149, and S153 into Eqn. S154 yields Eqn. S155.

$$C(t) = A_0 + \frac{-k_2A_0}{k_2 - k_1}exp(-k_1FGt) + A_0\left(\frac{k_1}{k_2 - k_1}\right)exp(-k_2FGt) \quad \text{(S155)}$$

5.0 Supplemental figures

5.1 Model fit figures

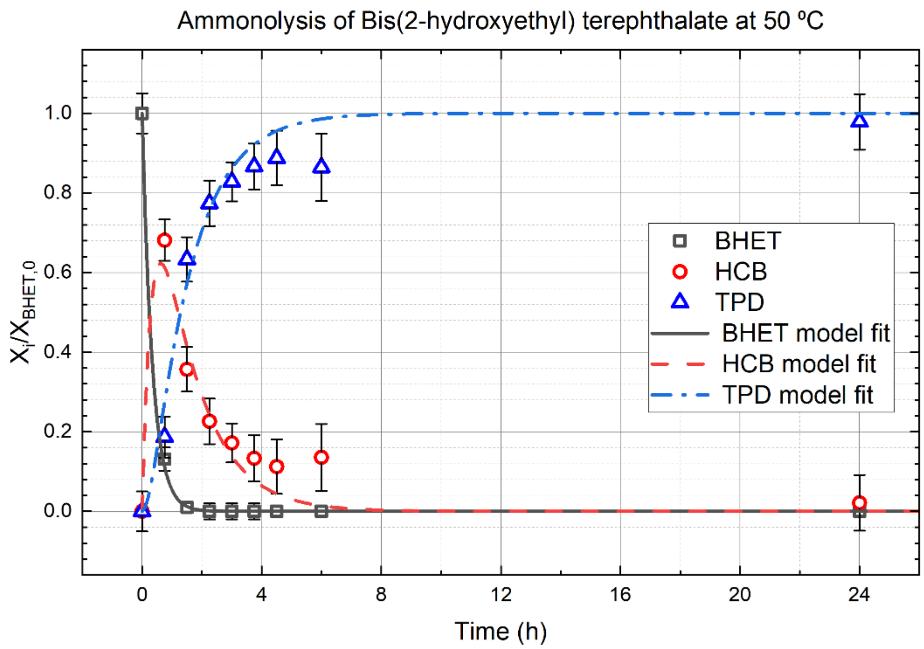

Figure S5. The ammonolysis of BHET with a set point of 50°C. The mole fraction date was plotted alongside the model fit. The reaction was conducted in 45 mol% ammonia dissolved in ethylene glycol. The k' values determined by this fit are 2.93 h^{-1} and 0.76 h^{-1} for reaction steps 1 and 2 respectively.

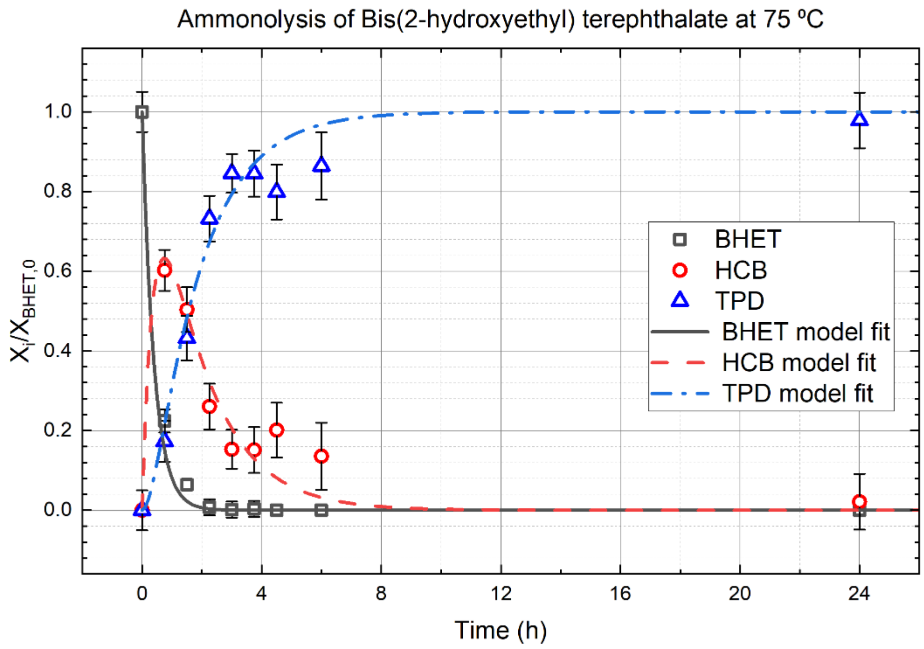

Figure S6. The ammonolysis of BHET at a temperature setpoint of 75 °C. The mole fraction was plotted alongside the model fit. The reaction was conducted in 22.5 mol% ammonia dissolved in ethylene glycol. The k' values determined by this fit are 2.53 h^{-1} and 0.62 h^{-1} for reaction steps 1 and 2 respectively.

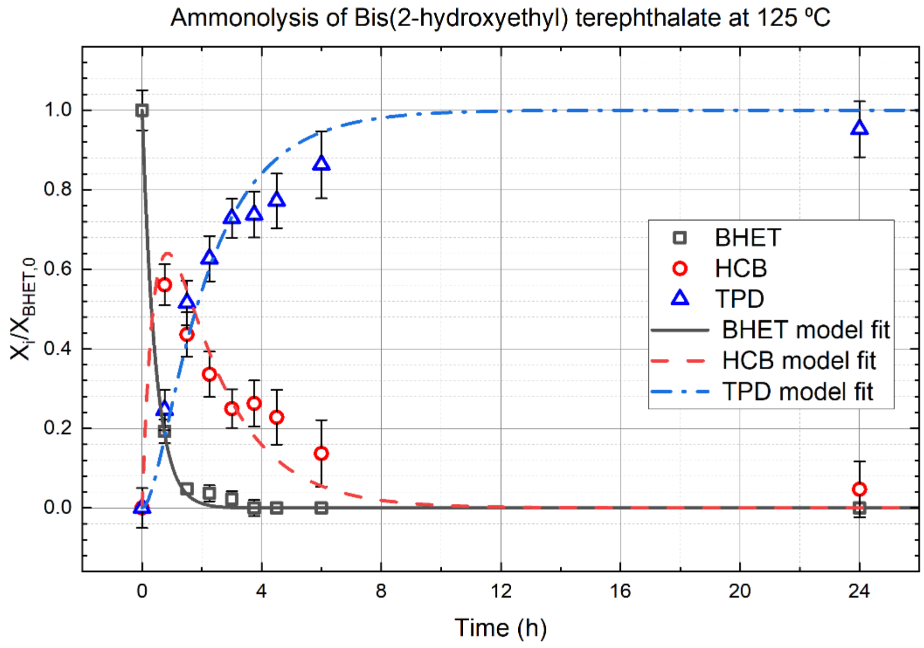

Figure S7. The ammonolysis of BHET at a temperature setpoint of 125 °C. The mole fraction was plotted alongside the model fit. The reaction was conducted in 11 mol% ammonia dissolved in ethylene glycol. The k' values determined by this fit are 2.24 h^{-1} and 0.53 h^{-1} for reaction steps 1 and 2 respectively.

5.2 Temperature ramp figures

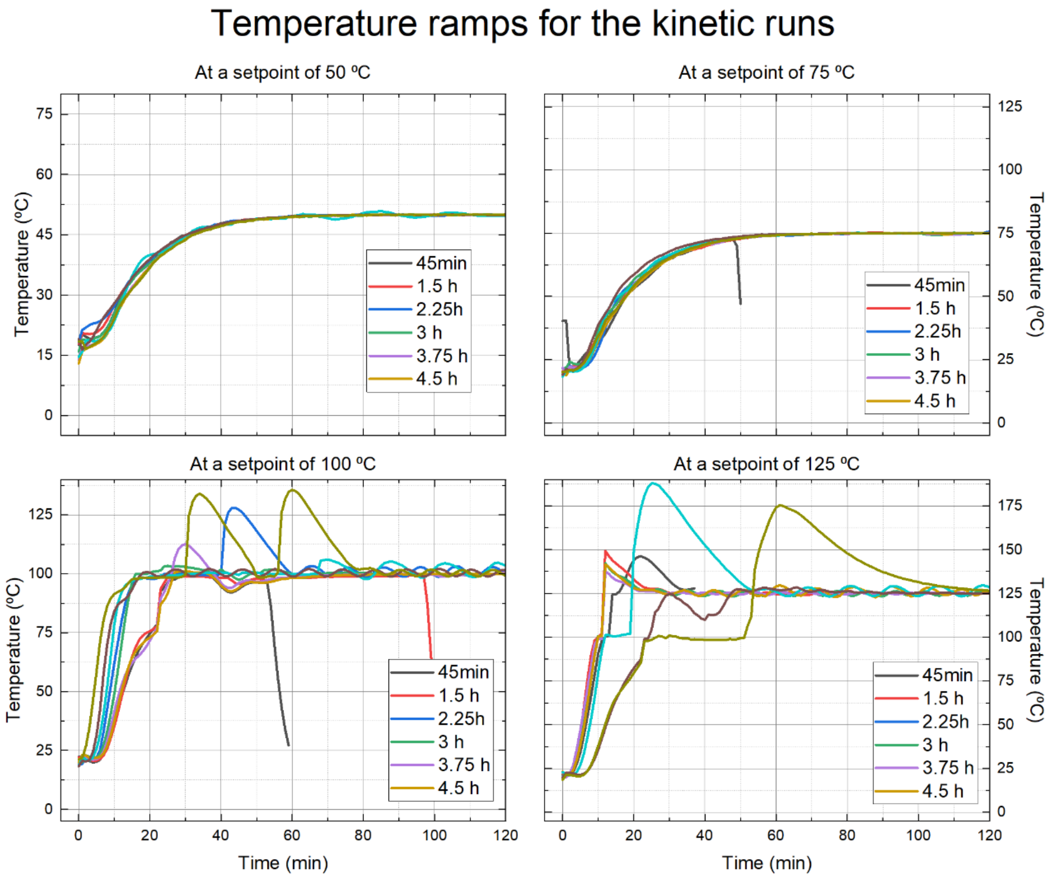

Figure S8. The temperature ramp for each timed run for the ammonolysis of BHET at 50°C. At this temperature there was little to no overshoot or oscillation. There is still error associated with the temperature ramp because the average temperature across 45 min of reaction time will always be less that the average across 24h. To mitigate the error, an average temperature was calculated for each run. The procedure for this calculation is further explained in this document.

5.3 NMR figures

5.3.1 BHET 50ºC proton NMRs

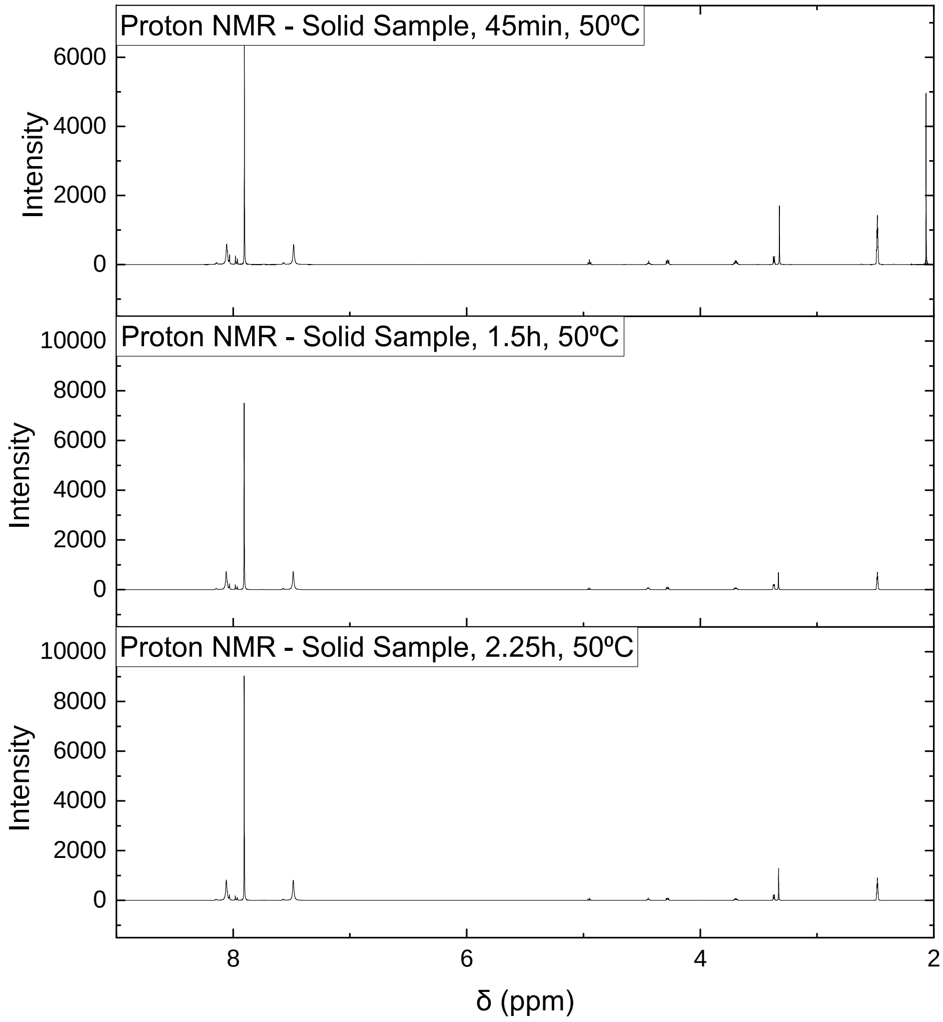

Figure S9. The Proton NMR for the solid samples taken after 45min,1.5h, and 2.25h of reaction at 50 ºC. The NMR was taken in DMSO-d6 on a 500 MHz NMR. The relaxation delay was set to 2 seconds and 32 scans were taken. After 2.25h the spectra was nearly identical to terephthalamide. The results of the NMR are as follows. Terephthalamide ^{1}H NMR ((500MHz, DMSO) δ 8.06 (s, 2H), 7.91 (s, 4H), 7.49 (s, 2H)), Bis(2hydroxyethyl) terephthalate ^{1}H NMR ((500 MHz, DMSO) δ 8.12 (s, 4H), 4.97 (t, J=5.7Hz, 2H),4.31(t, J=4.9Hz,2H), 3.71(q,J=5.7Hz, J=4.9Hz, 4)), and 2-hydroxyethyl 4-carbamoylbenzoate (1137-99-1) ^{1}H NMR ((500 MHz, DMSO) δ 8.14 (s, 1H), 8.07 – 8.02 (m, 2H), 8.00 – 7.95 (m, 2H), 7.57 (s, 1H), 4.95(t, J=5.7Hz, 1H), 4.31-4.25(m, 2H),3.73-3.66 (m, 2H)).

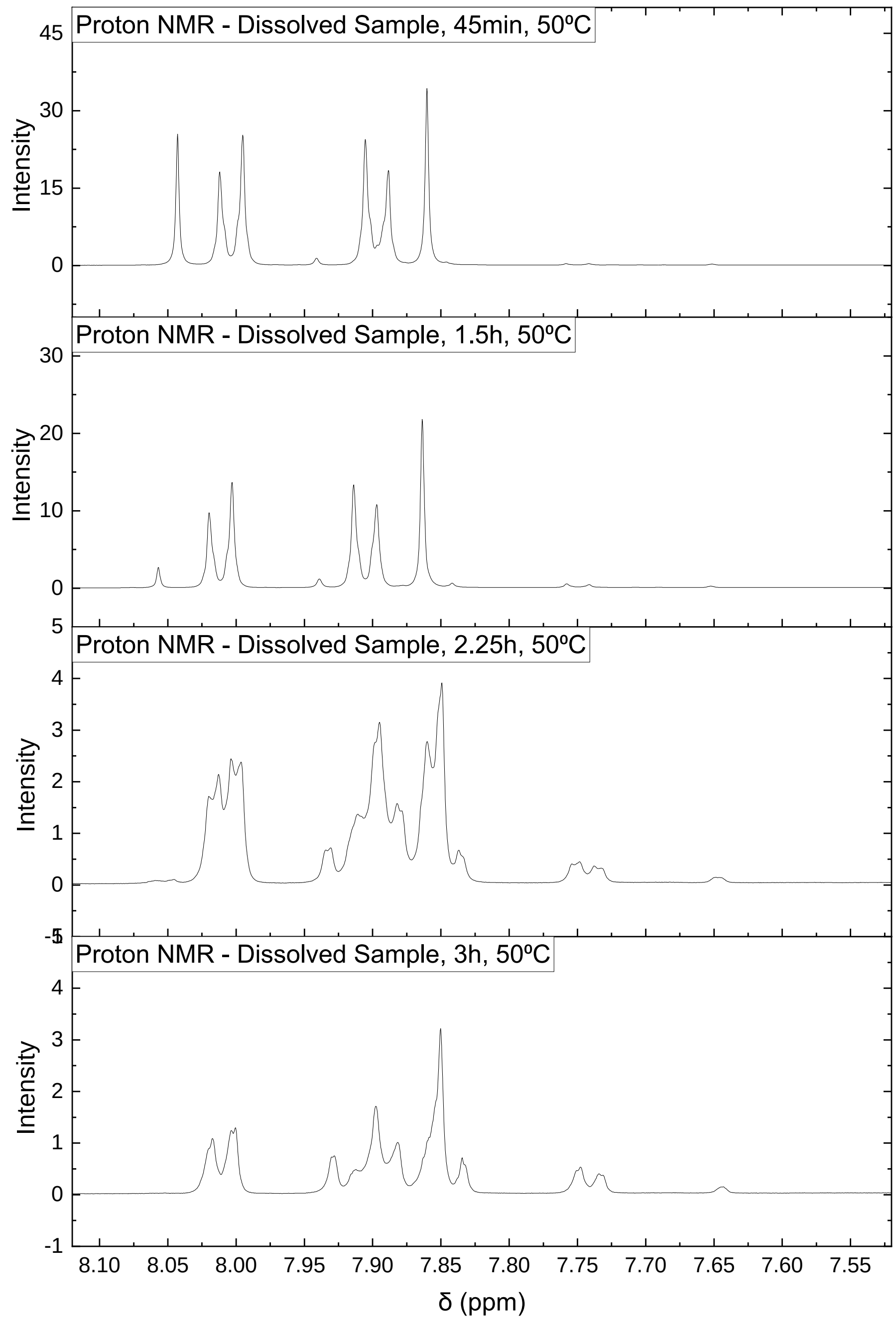

Figure S10. A proton NMR of the samples that remained dissolved in the ethylene glycol and ammonia solution after a reaction at 50 ^{0}C for 45min,1.5h, 2.25h and 3h. The NMR was taken in DMSO-d6 on a 500 MHz NMR. The relaxation delay was set to 2 seconds and 32 scans were taken. The peaks are shifted from their locations using just DMSO as the solvent. The aromatic peaks are BHET (8.04(s, 4H)), HCB (8.02-7.99(m, 2H), 7.92-7.88(m, 2H), TPD (7.86(s, 4H)). These peak locations are not perfectly consistent across all samples but they only change ±.05PPM and their order stays consistent.

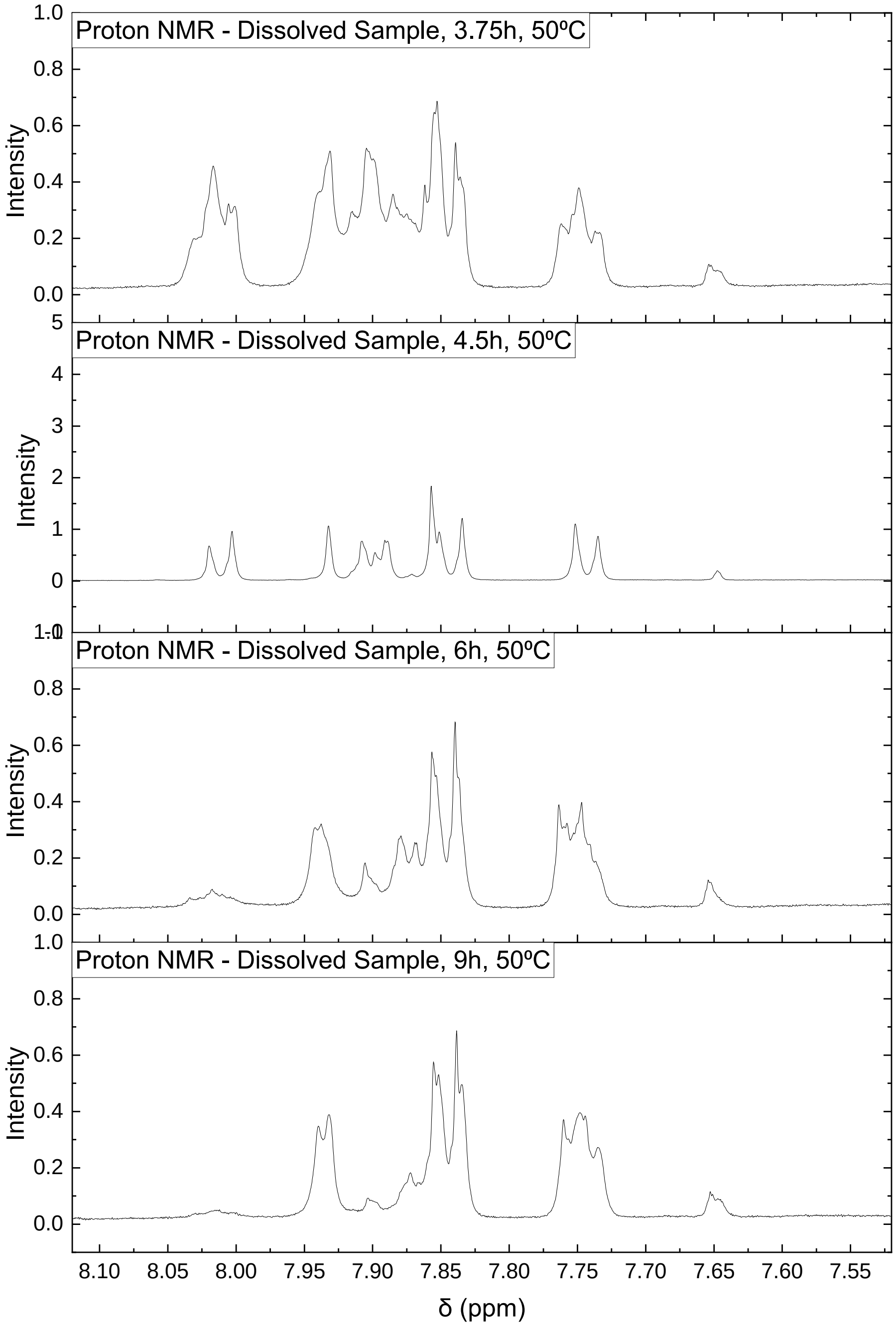

Figure S11. A proton NMR of the samples that remained dissolved in the ethylene glycol and ammonia solution after a reaction at 50 °C for 3.75h, 4.5h, 6h, 9h. The NMR was taken in DMSO-d6 on a 500 MHz NMR. The relaxation delay was set to 2 seconds and 32 scans were taken. The peaks are shifted from their locations using just DMSO as the solvent. The aromatic peaks are BHET (8.04(s, 4H)), HCB (8.02-7.99(m, 2H), 7.92-7.88(m, 2H), TPD (7.86(s, 4H)). These peak locations are not perfectly consistent across all samples but they only change ±.05PPM and their order stays consistent.

5.3.2 BHET 75°C proton NMRs

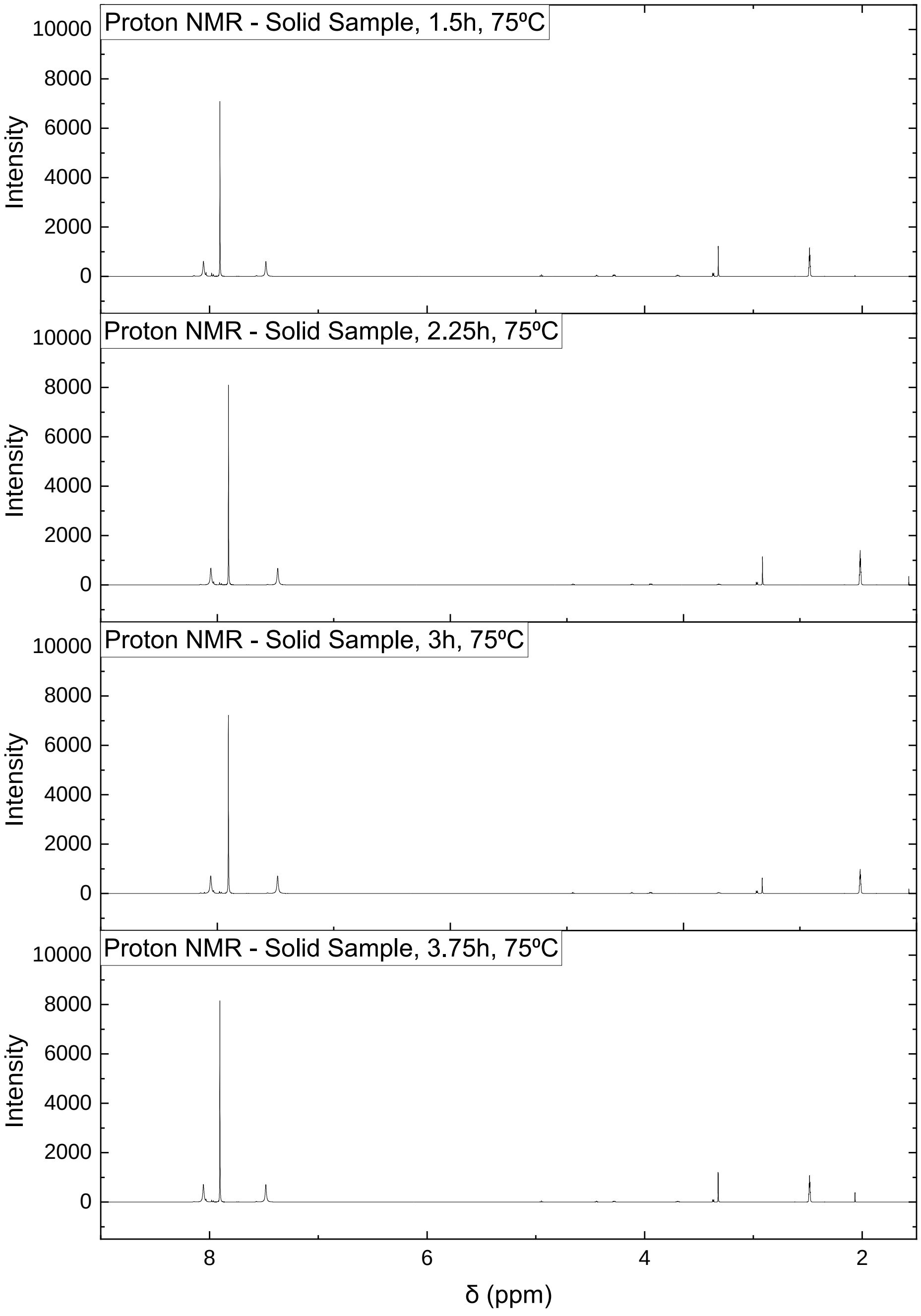

Figure S12. The Proton NMR spectra for the solid samples taken after 1.5h,2.25h, and 3.75h of reaction at 75 ^{o}C. The NMR was taken in DMSO-d6 on a 500 MHz NMR. The relaxation delay was set to 2 seconds and 32 scans were taken. The NMR spectra was mainly TPD after 3.75h. The results of the NMR are as follows. Terephthalamide ^{1}H NMR ((500MHz, DMSO) δ 8.06 (s, 2H), 7.91 (s, 4H), 7.49 (s, 2H)), Bis(2hydroxyethyl) terephthalate ^{1}H NMR ((500 MHz, DMSO) δ 8.12 (s, 4H), 4.97 (t, J=5.7Hz, 2H),4.31(t, J=4.9Hz,2H), 3.71(q,J=5.7Hz, J=4.9Hz, 4)), and 2-hydroxyethyl 4-carbamoylbenzoate (1137-99-1) ^{1}H NMR ((500 MHz, DMSO) δ 8.14 (s, 1H), 8.07 – 8.02 (m, 2H), 8.00 – 7.95 (m, 2H), 7.57 (s, 1H), 4.95(t, J=5.7Hz, 1H), 4.31-4.25(m, 2H),3.73-3.66 (m, 2H)).

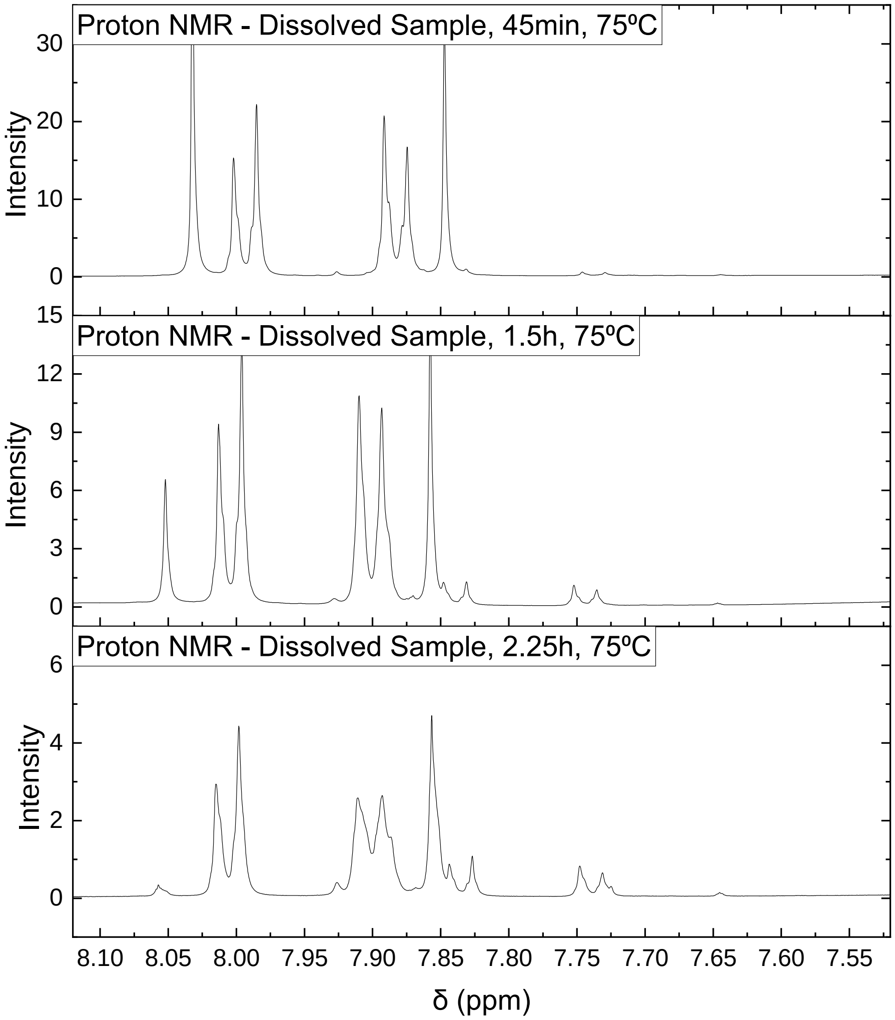

Figure S13. Proton NMR spectra of the samples that remained dissolved in the ethylene glycol and ammonia solution after a reaction at 75 ºC for 45min, 1.5h, and 2.25h. The NMR was taken in DMSO-d6 on a 500 MHz NMR. The relaxation delay was set to 2 seconds and 32 scans were taken. The peaks are shifted from their locations using just DMSO as the solvent. The aromatic peaks are BHET (8.04(s, 4H)), HCB (8.02-7.99(m, 2H), 7.92-7.88(m, 2H), TPD (7.86(s, 4H)). These peak locations are not perfectly consistent across all samples but they only change ±.05PPM and their order stays consistent.

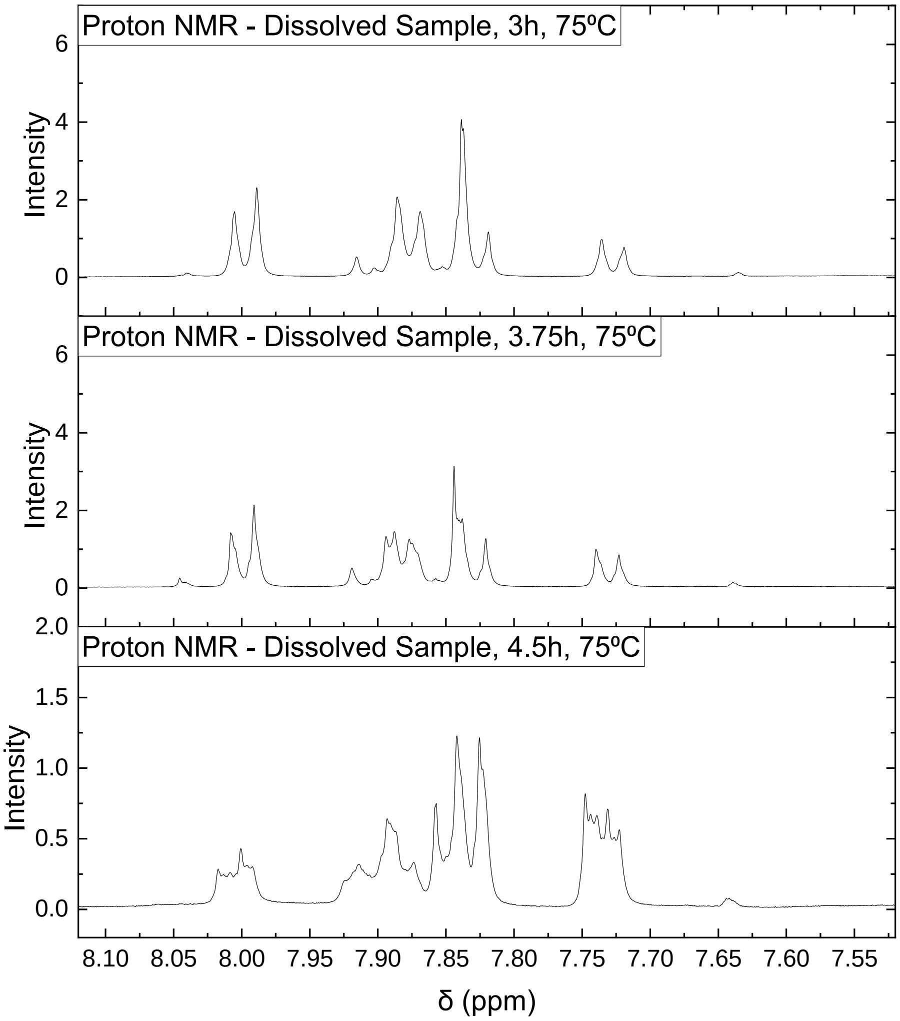

Figure S14. Proton NMR spectra of the samples that remained dissolved in the ethylene glycol and ammonia solution after a reaction at 75 ºC for 3h, 3.75h, and 4.5h. The NMR was taken in DMSO-d6 on a 500 MHz NMR. The relaxation delay was set to 2 seconds and 32 scans were taken. The peaks are shifted from their locations using just DMSO as the solvent. The aromatic peaks are BHET (8.04(s, 4H)), HCB (8.02-7.99(m, 2H), 7.92-7.88(m, 2H), TPD (7.86(s, 4H)). These peak locations are not perfectly consistent across all samples but they only change ±.05PPM and their order stays consistent.

5.3.3 BHET 100°C proton NMRs

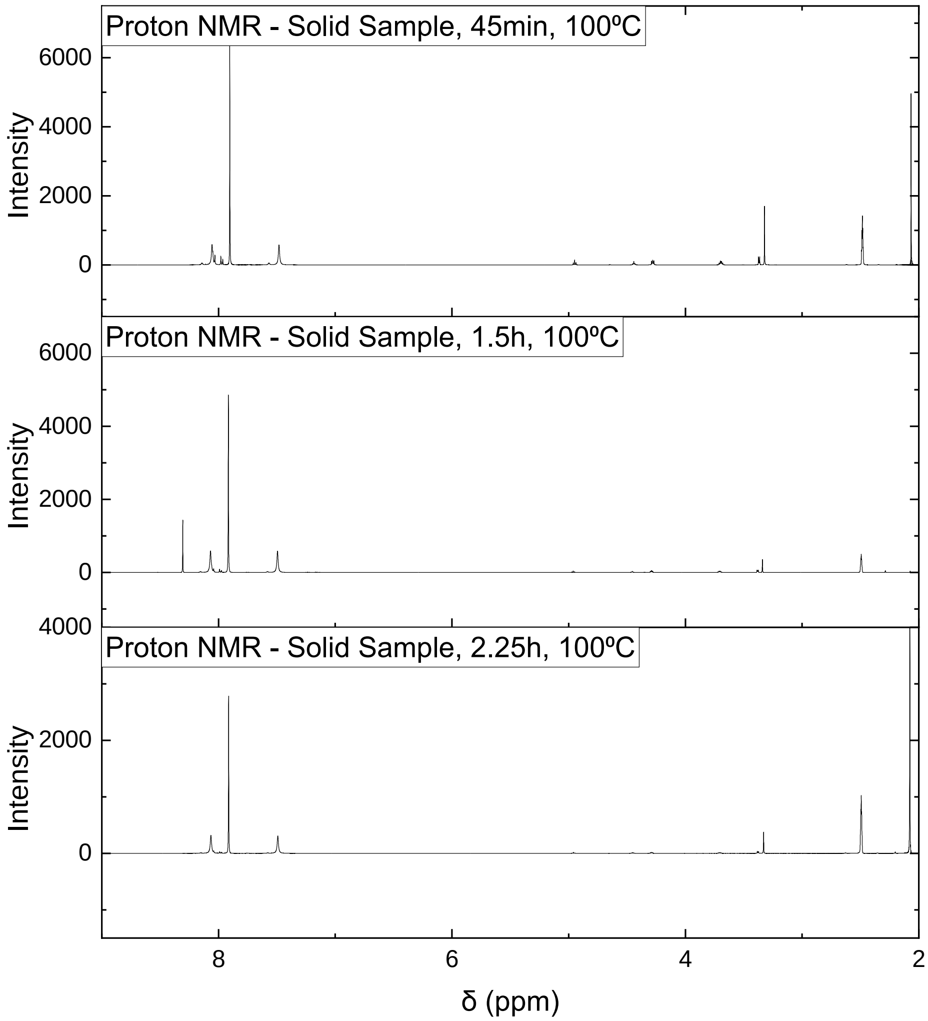

Figure S15. The Proton NMR spectra for the solid samples taken after 45min, 1.5h, and 2.25h of reaction at 100°C. The NMR was taken in DMSO-d6 on a 500 MHz NMR. The relaxation delay was set to 2 seconds and 32 scans were taken. The spectra closely followed that of TPD after 2.25 h. The results of the NMR are as follows. Terephthalamide ^{1}H NMR ((500MHz, DMSO) δ 8.06 (s, 2H), 7.91 (s, 4H), 7.49 (s, 2H)), Bis(2hydroxyethyl) terephthalate ^{1}H NMR ((500 MHz, DMSO) δ 8.12 (s, 4H), 4.97 (t, J=5.7Hz, 2H),4.31(t, J=4.9Hz,2H), 3.71(q,J=5.7Hz, J=4.9Hz, 4)), and 2-hydroxyethyl 4-carbamoylbenzoate (1137-99-1) ^{1}H NMR ((500 MHz, DMSO) δ 8.14 (s, 1H), 8.07 – 8.02 (m, 2H), 8.00 – 7.95 (m, 2H), 7.57 (s, 1H), 4.95(t, J=5.7Hz, 1H), 4.31-4.25(m, 2H),3.73-3.66 (m, 2H)).

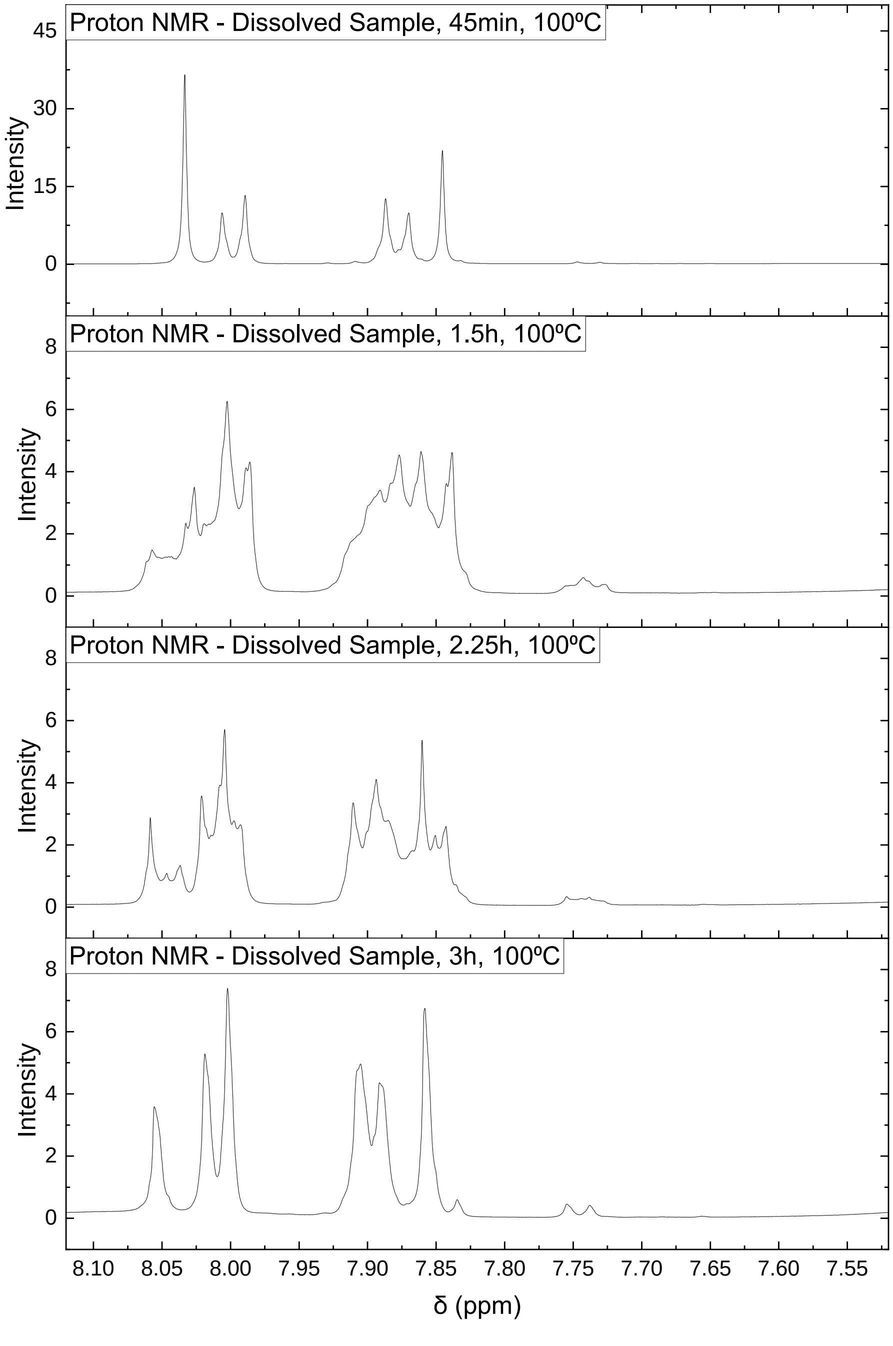

Figure S16. Proton NMR spectra of the samples that remained dissolved in the ethylene glycol and ammonia solution after a reaction at 100 ºC for 45 min, 1.5h, 2.25h, and 3h. The NMR was taken in DMSO-d6 on a 500 MHz NMR. The relaxation delay was set to 2 seconds and 32 scans were taken. The peaks are shifted from their locations using just DMSO as the solvent. The aromatic peaks are BHET (8.04(s, 4H)), HCB (8.02-7.99(m, 2H), 7.92-7.88(m, 2H), TPD (7.86(s, 4H)). These peak locations are not perfectly consistent across all samples but they only change ±.05PPM and their order stays consistent.

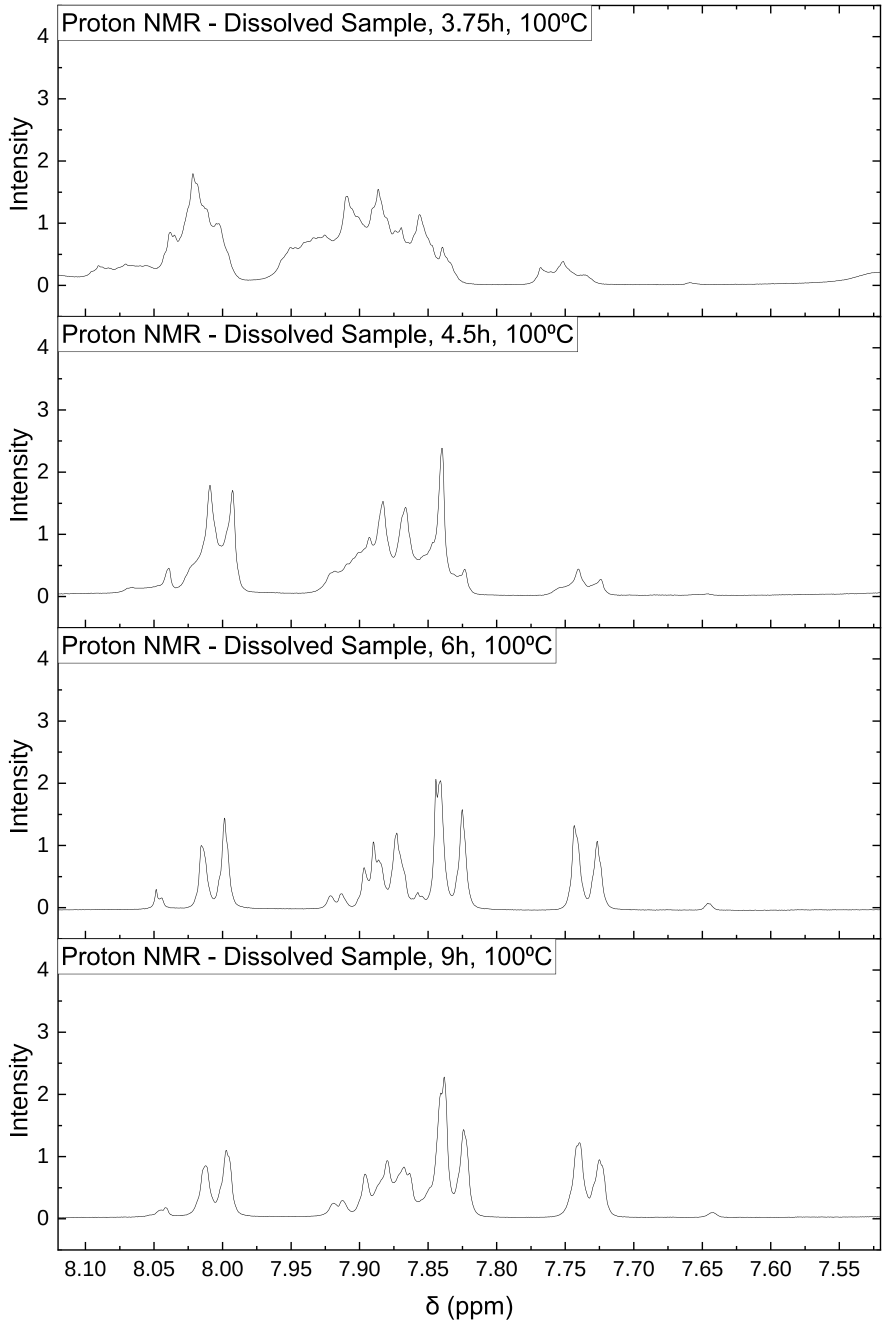

Figure S17. Proton NMR spectra of the samples that remained dissolved in the ethylene glycol and ammonia solution after a reaction at 100 ^{0}C for 3.75h, 4.5h, 6h and 9h. The NMR was taken in DMSO-d6 on a 500 MHz NMR. The relaxation delay was set to 2 seconds and 32 scans were taken. The peaks are shifted from their locations using just DMSO as the solvent. The aromatic peaks are BHET (8.04(s, 4H)), HCB (8.02-7.99(m, 2H), 7.92-7.88(m, 2H), TPD (7.86(s, 4H)). These peak locations are not perfectly consistent across all samples but they only change ±.05PPM and their order stays consistent.

5.3.4 BHET 125°C proton NMRs

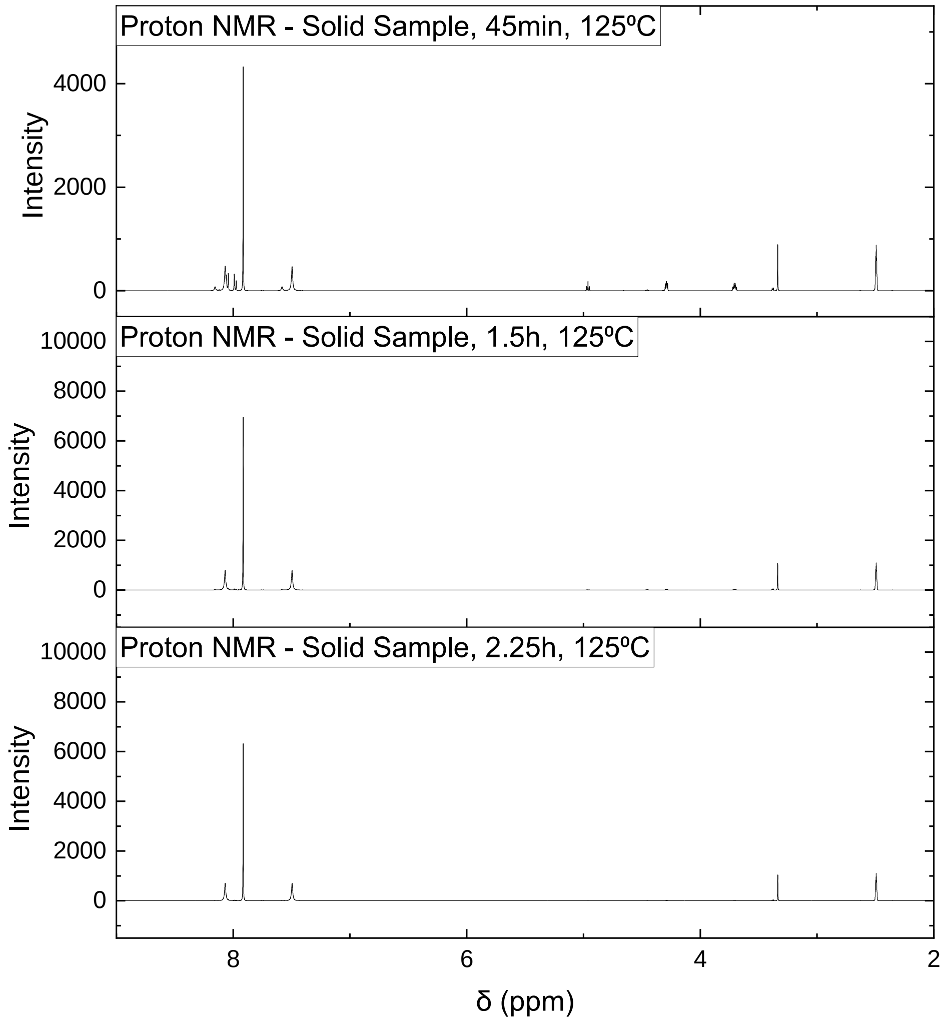

Figure S18. The Proton NMR spectra for the solid samples taken after 45min, 1.5h, and 2.25h of reaction at 125°C. The NMR was taken in DMSO-d6 on a 500 MHz NMR. The relaxation delay was set to 2 seconds and 32 scans were taken. The spectra closely followed that of TPD after 2.25 h. The results of the NMR are as follows. Terephthalamide ^{1}H NMR ((500MHz, DMSO) δ 8.06 (s, 2H), 7.91 (s, 4H), 7.49 (s, 2H)), Bis(2hydroxyethyl) terephthalate ^{1}H NMR ((500 MHz, DMSO) δ 8.12 (s, 4H), 4.97 (t, J=5.7Hz, 2H),4.31(t, J=4.9Hz,2H), 3.71(q,J=5.7Hz, J=4.9Hz, 4)), and 2-hydroxyethyl 4-carbamoylbenzoate (1137-99-1) ^{1}H NMR ((500 MHz, DMSO) δ 8.14 (s, 1H), 8.07 – 8.02 (m, 2H), 8.00 – 7.95 (m, 2H), 7.57 (s, 1H), 4.95(t, J=5.7Hz, 1H), 4.31-4.25(m, 2H),3.73-3.66 (m, 2H)).

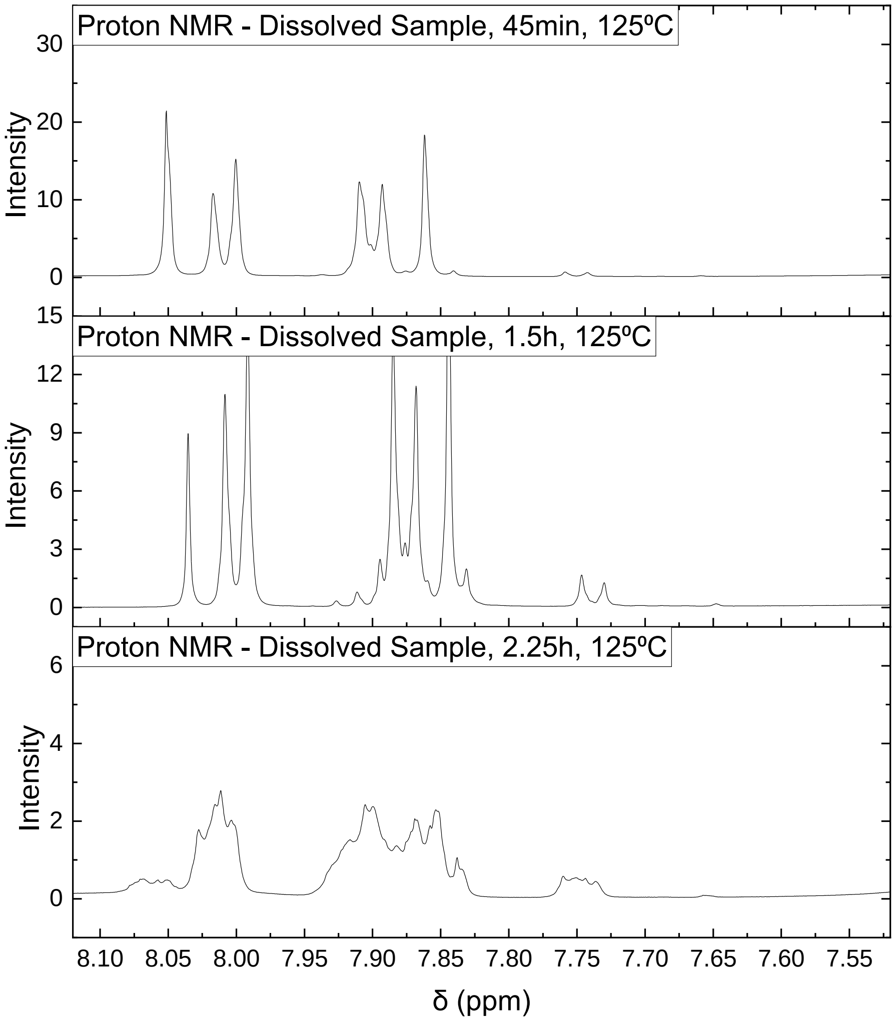

Figure S19. Proton NMR spectra of the samples that remained dissolved in the ethylene glycol and ammonia solution after a reaction at 100 ^{0}C for 3.75h, 4.5h, 6h and 9h. The NMR was taken in DMSO-d6 on a 500 MHz NMR. The relaxation delay was set to 2 seconds and 32 scans were taken. The peaks are shifted from their locations using just DMSO as the solvent. The aromatic peaks are BHET (8.04(s, 4H)), HCB (8.02-7.99(m, 2H), 7.92-7.88(m, 2H), TPD (7.86(s, 4H)). These peak locations are not perfectly consistent across all samples but they only change ±.05PPM and their order stays consistent.

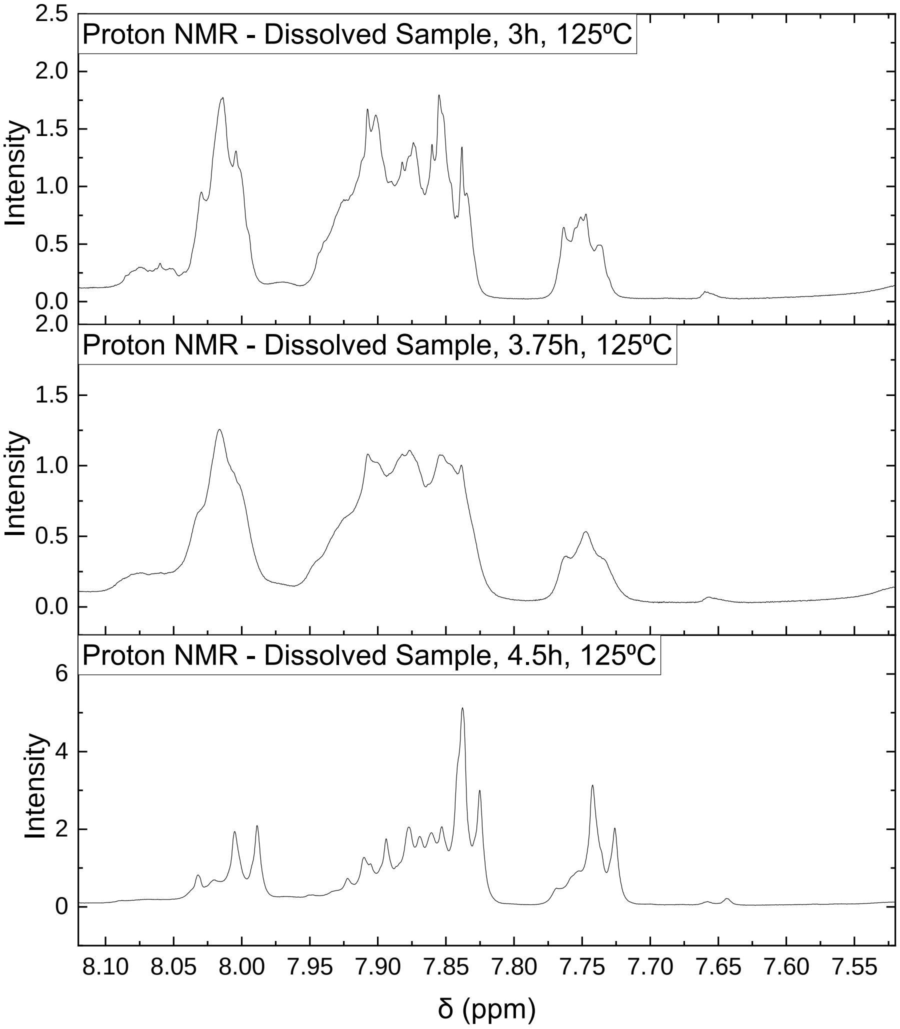

Figure S20. Proton NMR spectra of the samples that remained dissolved in the ethylene glycol and ammonia solution after a reaction at 100 ^{0}C for 3.75h, 4.5h, 6h and 9h. The NMR was taken in DMSO-d6 on a 500 MHz NMR. The relaxation delay was set to 2 seconds and 32 scans were taken. The peaks are shifted from their locations using just DMSO as the solvent. The aromatic peaks are BHET (8.04(s, 4H)), HCB (8.02-7.99(m, 2H), 7.92-7.88(m, 2H), TPD (7.86(s, 4H)). These peak locations are not perfectly consistent across all samples but they only change ±.05PPM and their order stays consistent.

5.3.5 Proton NMRs of oligomeric PET

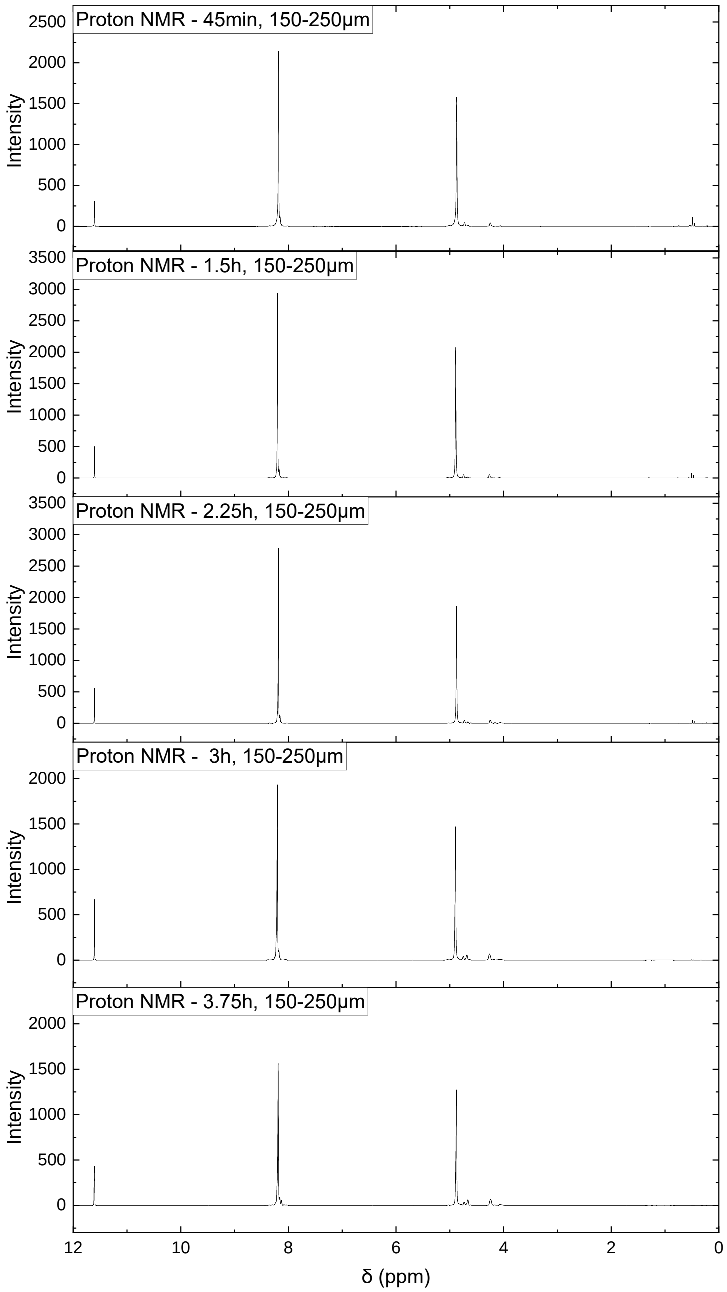

Figure S21. The Proton NMR for the PET (150-250μm) sample taken after 45min of reaction at 100°C. The NMR was taken in trifluoracetic acid on a 500 MHz NMR. The relaxation delay was set to 2 seconds and 32 scans were taken. PET has aromatic protons (8.20ppm), aliphatic protons (4.88ppm), and the protons on the end-groups (small peaks between 4.74-4.08ppm).

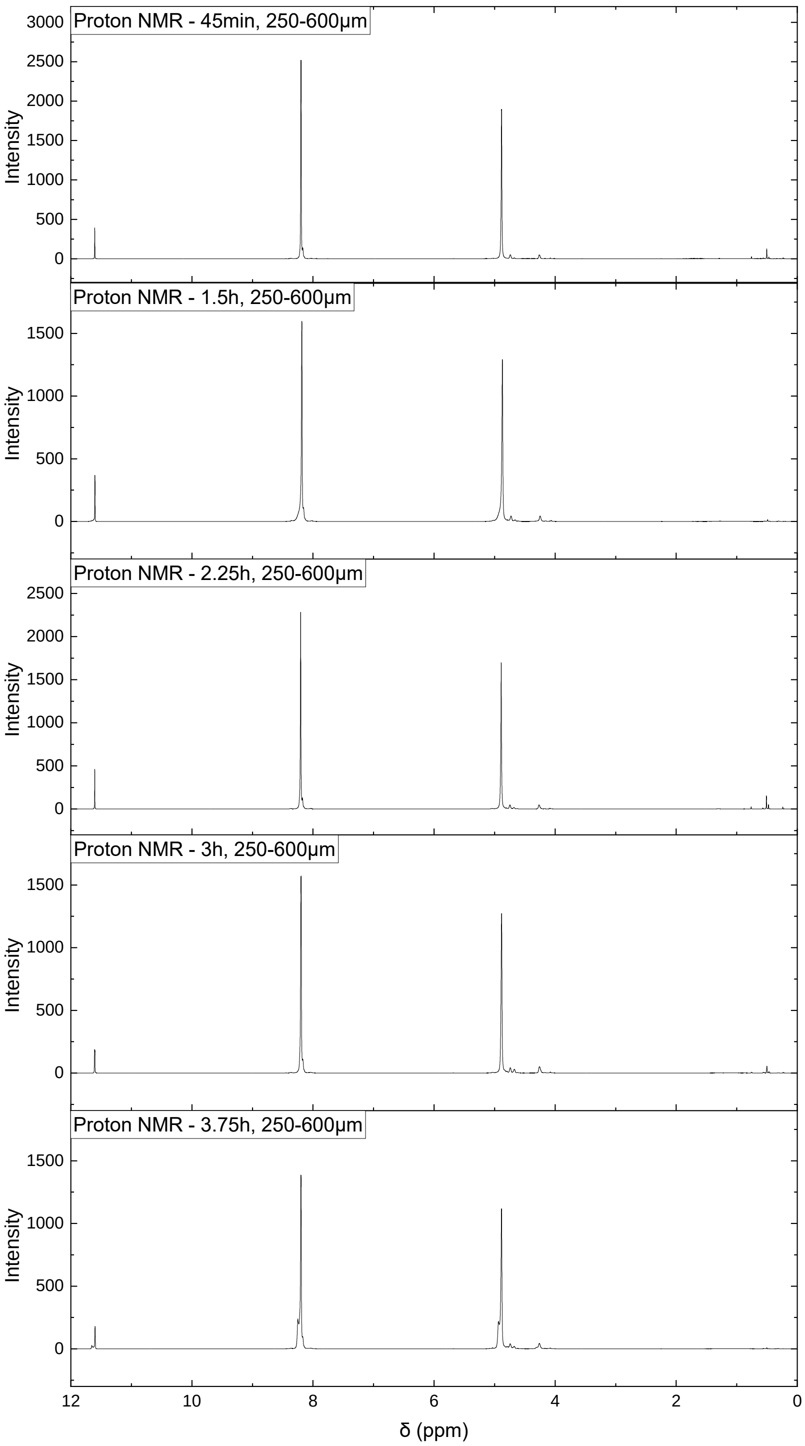

Figure S22. The Proton NMR for the PET (250-600µm) sample taken after 45min of reaction at 100°C. The NMR was taken in trifluoracetic acid on a 500 MHz NMR. The relaxation delay was set to 2 seconds and 32 scans were taken. PET has aromatic protons (8.20ppm), aliphatic protons (4.88ppm), and the protons on the end-groups (small peaks between 4.74-4.08ppm).

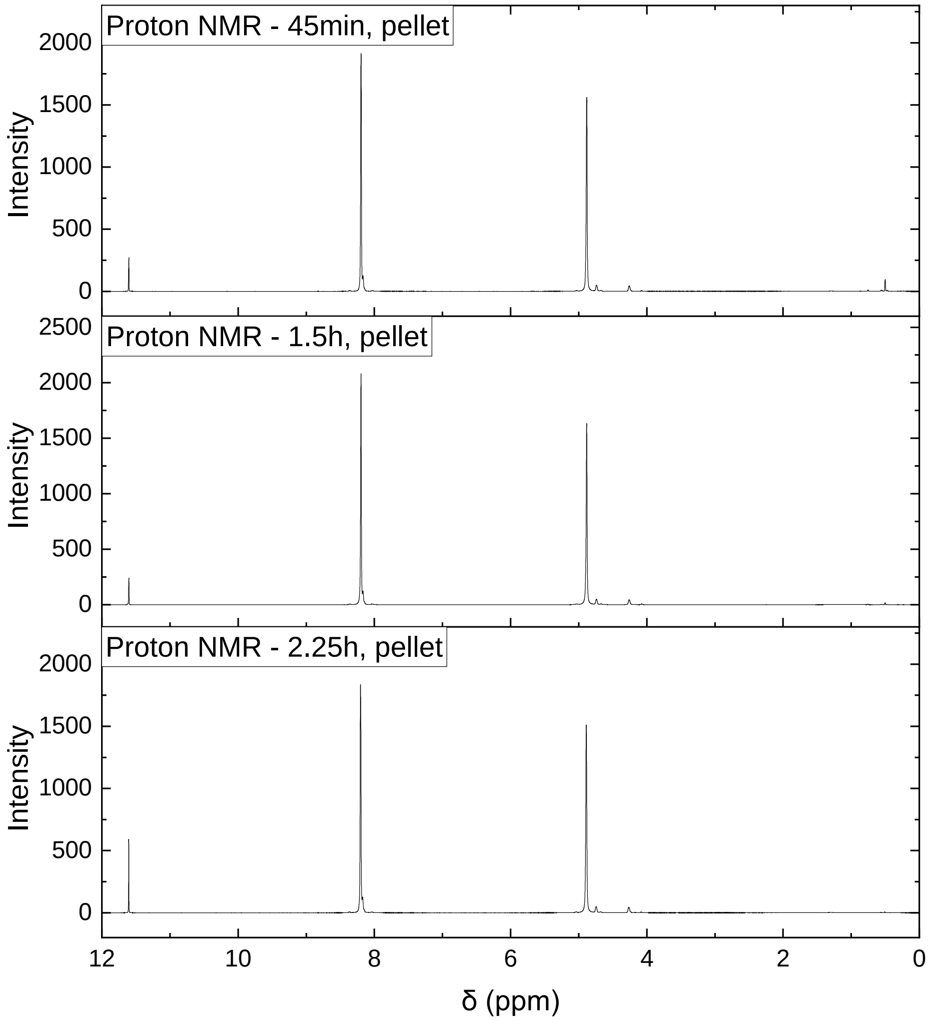

Figure S23. The Proton NMR for the PET (pellet, 1800-2500μm) sample taken after 45min of reaction at 100°C. The NMR was taken in trifluoracetic acid on a 500 MHz NMR. The relaxation delay was set to 2 seconds and 32 scans were taken. PET has aromatic protons (8.20ppm), aliphatic protons (4.88ppm), and the protons on the end-groups (small peaks between 4.74-4.08ppm).

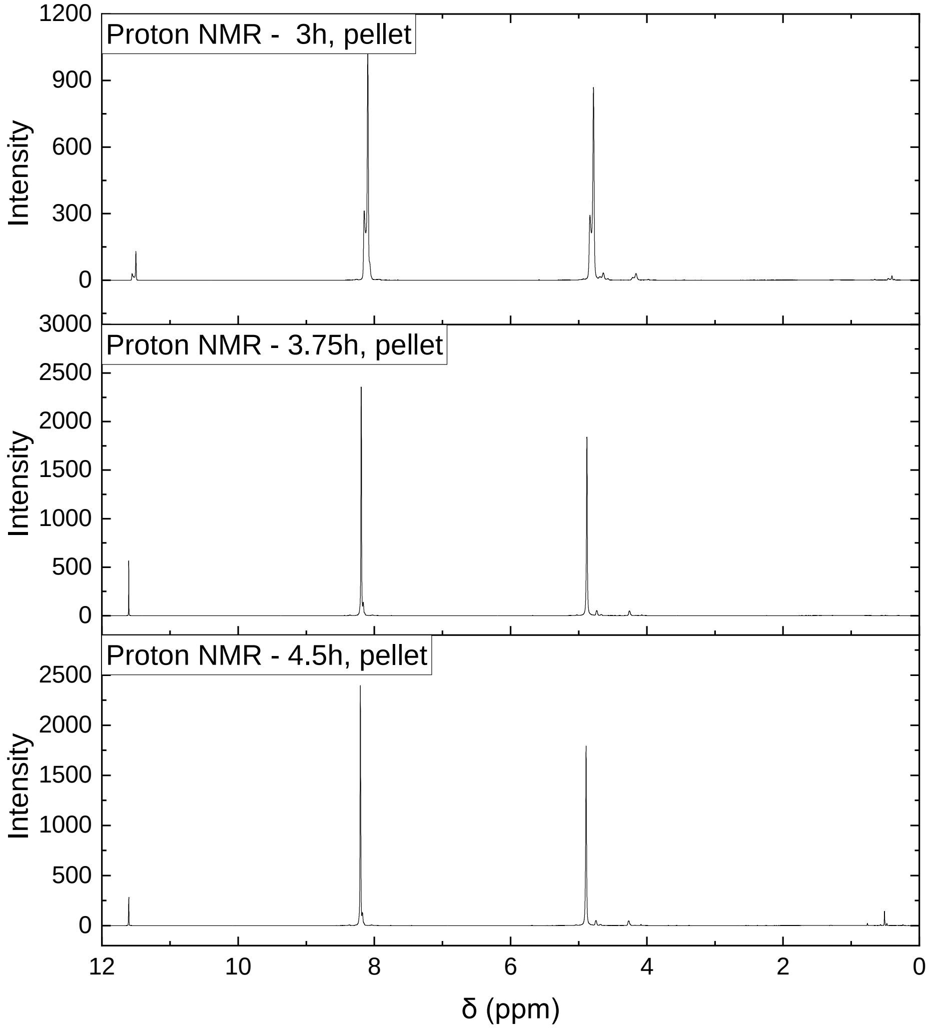

Figure S24. The Proton NMR for the PET (pellet, 1800-2500µm) sample taken after 45min of reaction at 100°C. The NMR was taken in trifluoracetic acid on a 500 MHz NMR. The relaxation delay was set to 2 seconds and 32 scans were taken. PET has aromatic protons (8.20ppm), aliphatic protons (4.88ppm), and the protons on the end-groups (small peaks between 4.74-4.08ppm).

5.3.6 Proton NMRs of PET thermoforms undergoing ammonolysis

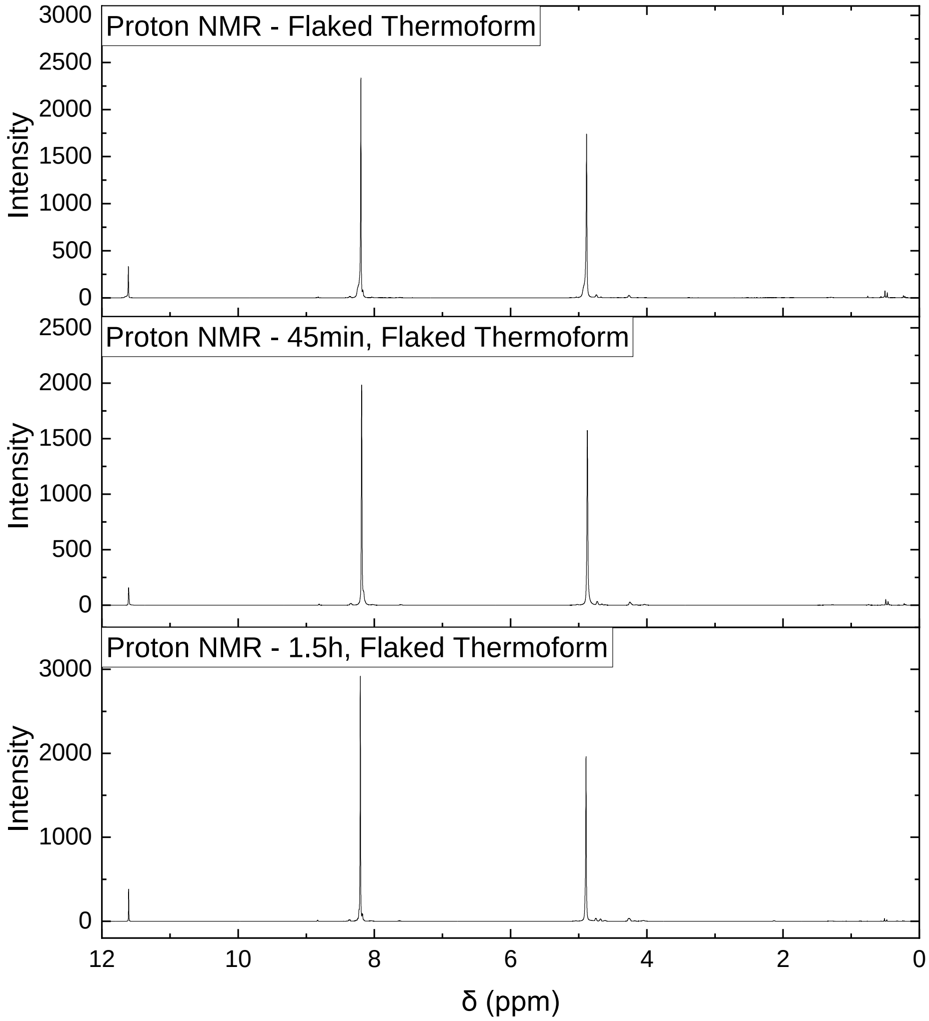

Figure S25. The Proton NMR for the PET (Thermoform, flaked) sample taken after 45min of reaction at 100 °C. The NMR was taken in trifluoracetic acid on a 500 MHz NMR. The relaxation delay was set to 2 seconds and 32 scans were taken. PET has aromatic protons (8.20ppm), aliphatic protons (4.88ppm), and the protons on the end-groups (small peaks between 4.74-4.08ppm).

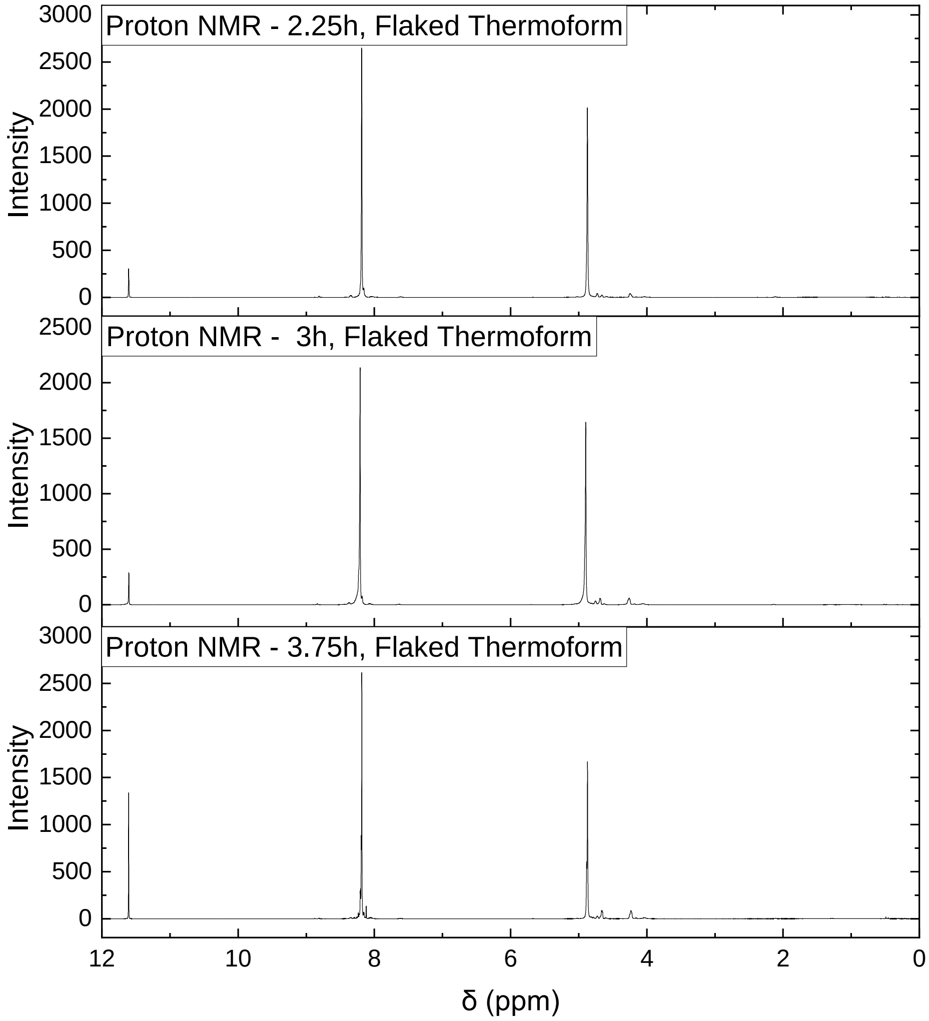

Figure S26. The Proton NMR for the PET (Thermoform, flaked) sample taken after 45min of reaction at 100°C. The NMR was taken in trifluoracetic acid on a 500 MHz NMR. The relaxation delay was set to 2 seconds and 32 scans were taken. PET has aromatic protons (8.20ppm), aliphatic protons (4.88ppm), and the protons on the end-groups (small peaks between 4.74-4.08ppm).

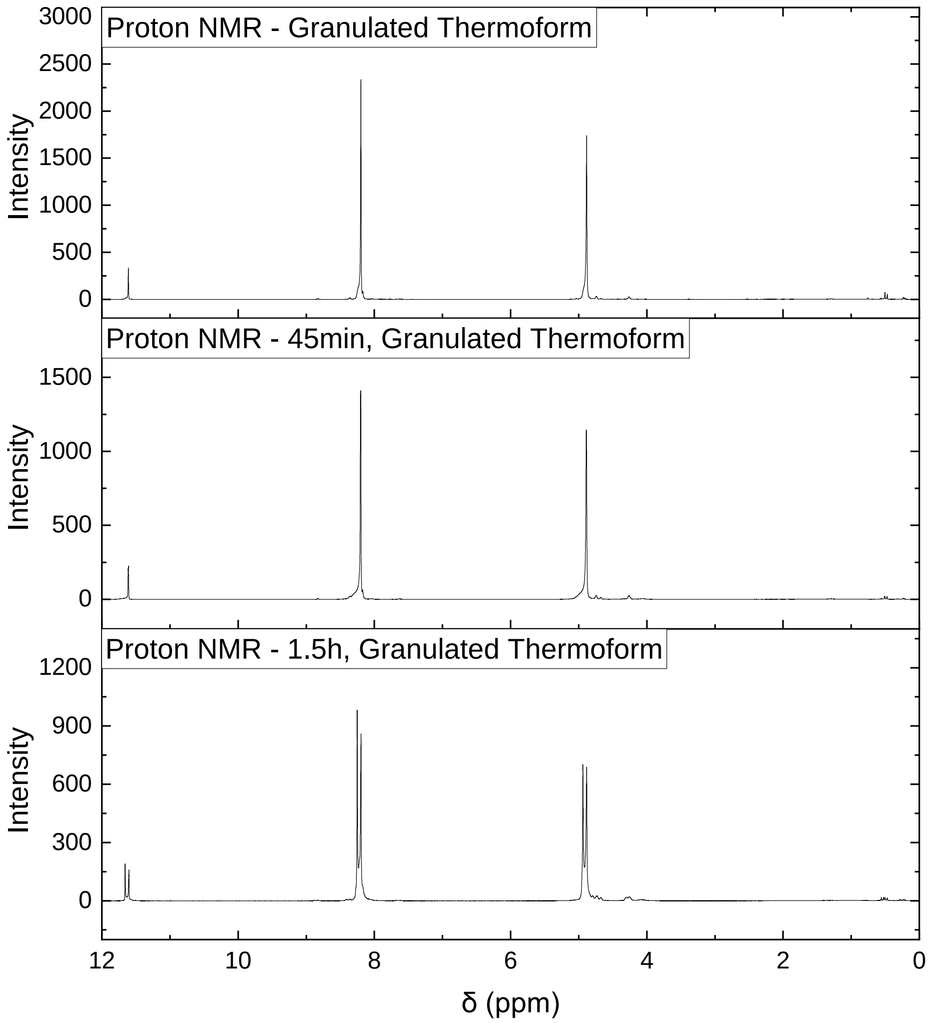

Figure S27. The Proton NMR for the PET (Thermoform, 150-250μm) sample taken after 45min of reaction at 100°C. The NMR was taken in trifluoracetic acid on a 500 MHz NMR. The relaxation delay was set to 2 seconds and 32 scans were taken. PET has aromatic protons (8.20ppm), aliphatic protons (4.88ppm), and the protons on the end-groups (small peaks between 4.74-4.08ppm).

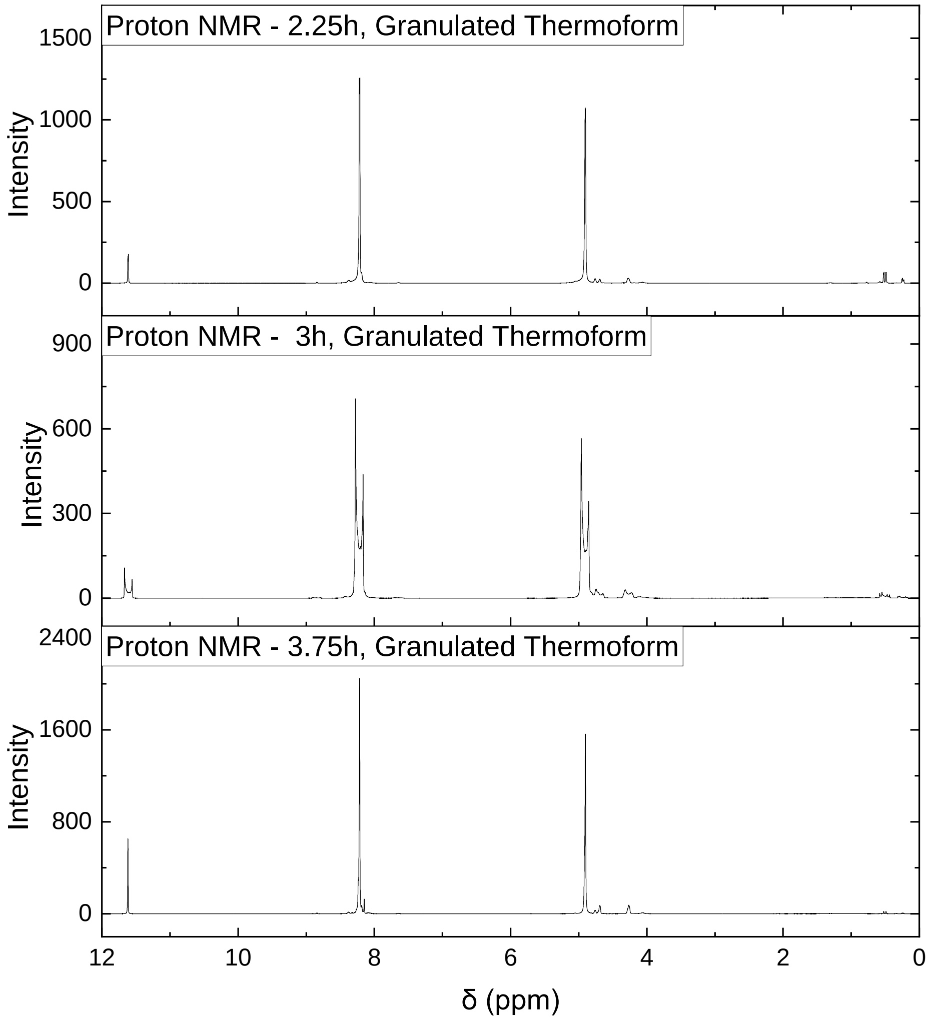

Figure S28. The Proton NMR for the PET (Thermoform, 150-250μm) sample taken after 45min of reaction at 100°C. The NMR was taken in trifluoracetic acid on a 500 MHz NMR. The relaxation delay was set to 2 seconds and 32 scans were taken. PET has aromatic protons (8.20ppm), aliphatic protons (4.88ppm), and the protons on the end-groups (small peaks between 4.74-4.08ppm).

5.4 Activity coefficients from different thermodynamic models

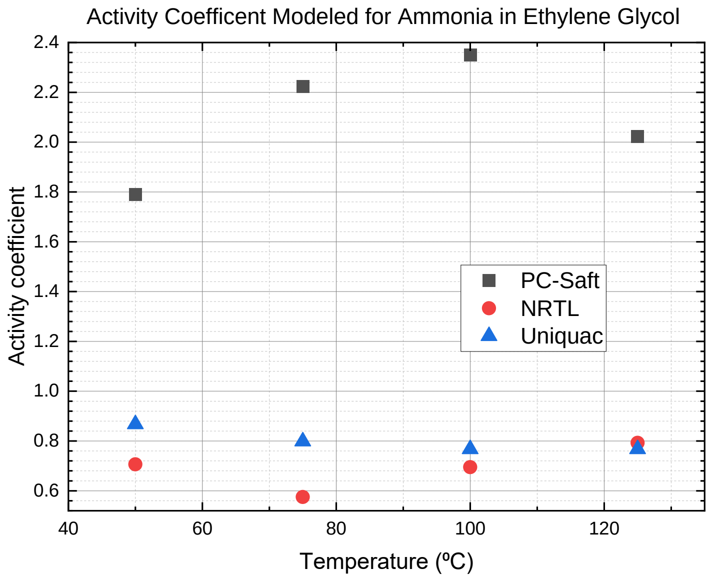

Figure S29. The activity coefficient for ammonia in ethylene glycol at the molar concentrations used in this work (45 mol%, 22mol%, 11mol%, and 11mol% for 50ºC, 75ºC, 100ºC and 125ºC respectively). Values estimated using ASPEN Plus. Applying the values obtained to the kinetic model described in this work does not improve the goodness of fit. The PC-SAFT activity model is data dependent and considered to be anomalous.

5.5 Crystallinity vs. Time

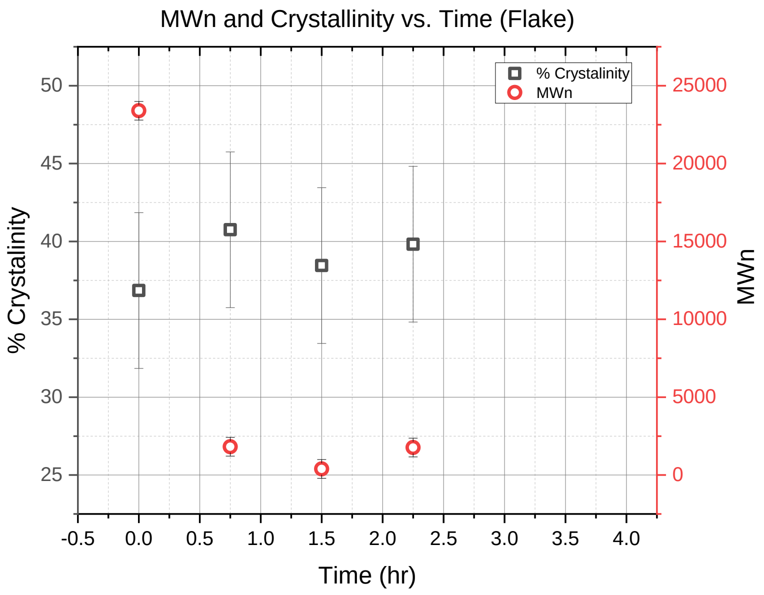

Figure S30. The number average molecular weight and percent crystallinity as a function of time for thermoform PET flake. The crystallinity was determined via differential scanning calorimetry (DSC). Overall it seems that despite a change in molecular weight, the percent crystallinity remained constant for this sample.

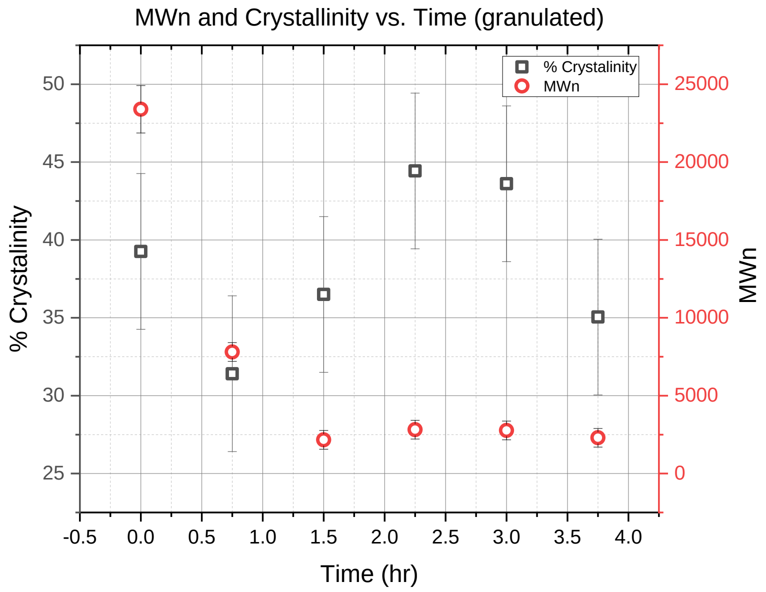

Figure S31. The number average molecular weight and percent crystallinity as a function of time for thermoform PET flake. The crystallinity was determined via differential scanning calorimetry (DSC). Overall it seems that the percent crystallinity increased and then decreased which could indicate that amorphous regions were preferentially reacted.

6.0 Python Example Code

```
import numpy as np
import matplotlib.pyplot as plt
from scipy.optimize import curve_fit,least_squares
# the kinetic model calculated analytically using laplace transforms
def model1(X, A1, Ea1):
   t,T,A0=X
   DMT0=.008315
   R=8.314
   k1=A1*A0*np.exp(-Ea1/R/T)
   return -k1*t
def model2(X, A2, Ea2):
   t,T, A0=X
   bhet0=.008315
   R=8.314
   k1=popt[0]*A0*np.exp(-popt[1]/R/T)
   k2=A2*A0*np.exp(-Ea2/R/T)
   return (1+(k2/(k1-k2))*np.exp(-k1*t)-(k1/(k1-k2))*np.exp(-k2*t))
time=np.array([.75,1.5,2.25,3,3.75,4.5,6,9,24,\
          .75,1.5,2.25,3,3.75,4.5,6,9,24,\
          .75,1.5,2.25,3,3.75,4.5,6,9,24,\
          .75,1.5,2.25,3,3.75,4.5,6,9,24])
Tavg=np.array([36.91,43.43,45.74,46.47,47.02,47.46,48.23,48.89,49.53,\
```

```
            53.40,62.97,66.97,69.29,70.08,70.86,72.03,73.26,74.23,\
            93.93,104.25,96.69,95.30,101.43,100.48,99.37,99.04,100.59,\
            106.53,119.32,120.24,121.57,121.72,123.10,122.87,122.85,124.44])
Tavg=Tavg+273.15

#A0 differs between setpoint T .45 for T=50, .225 for T=75 and .1125 for T=100 and 125
A0=np.array([.45,.45,.45,.45,.45,.45,.45,.45,.45,\
          .225,.225,.225,.225,.225,.225,.225,.225,.225,\
          .1125,.1125,.1125,.1125,.1125,.1125,.1125,.1125,.1125,\
          .1125,.1125,.1125,.1125,.1125,.1125,.1125,.1125,.1125])
#mole fraction data
bhet=np.array([.131689524,.010400702,\
           .224478038,.064241864,.007447585,.00163654,.003600968,\
           .294538188,.083913591,.047152057,.079256421,.023451245,.016070358,\
           .192604997,.048246719,.036971434,.022266804])
mono=np.array([.681375876,.356715236,.226227056,.172133652,.133326835,.112454566,.135568293,
.1930843,.021499363,\

.602406723,.503691808,.2604468992,.1529996004,.151386612,.201257746,.135568293,.1930843,.021
499363,\

.514250617,.560607578,.571974293,.486529999,.399760144,.298750272,.158644925,.22979411,.0214
99363,\

.561177706,.436117191,.336120963,.249787656,.262599189,.228089284,.136958122,.206469971,0])
tpd=np.array([.186935,.632884,.773773,.827866,.866673,.887545,.864432,.806916,.978501,\
          .173115,.432066,.732083,.845367,.845013,.798742,.864432,.806916,.978501,\
          .191211,.355479,.380874,.434214,.576789,.685179,.841355,.770206,.978501,\
          .246217,.515636,.626908,.727946,.737401,.771911,.863042,.79353,1])
y=np.array([time, Tavg, A0])
# #print(y)
# #take away the extra zeros for bhet
timebhet=np.array([.75,1.5,\
              .75,1.5,2.25,3,3.75,\
              .75,1.5,2.25,3,3.75,4.5,\
              .75,1.5,2.25,3])
Tavgbhet=np.array([36.91,43.43,\
             53.40,62.97,66.97,69.29,70.08,\
             93.93,104.25,96.69,95.30,101.43,100.48,\
             106.53,119.32,120.24,121.57])
Tavgbhet=Tavgbhet+273.15

A0bhet=np.array([.45,.45,\
          .225,.225,.225,.225,.225,\
          .1125,.1125,.1125,.1125,.1125,.1125,\
          .1125,.1125,.1125,.1125])

ybhet=np.array([timebhet, Tavgbhet, A0bhet])
```

```
# A0=.250597
logbhet=np.log(bhet)

guess=np.array([4000,25000])
popt,pcov=curve_fit(model1,ybhet,logbhet,p0=guess)
A1=popt
print(popt)
print(popt[0]*np.exp(-popt[1]/8.314/373.15))

popt2,pcov2=curve_fit(model2,y,tpa,p0=guess)
A2=popt2
print(popt2)
print(popt2[0]*np.exp(-popt2[1]/8.314/373.15))

#For error analysis sample a new set of data 50000  times
i=0
err=0
myEAsamples = []
myEAsamples2 = []
myAsamples = []
myAsamples2 = []
# #all temps
for _ in range(50000):
#because the data is time sensitive, randomly removing up to a fourth of the data without replacement makes the most sense
x=np.random.choice(np.arange(17),size=np.random.choice(4),replace=False)
   timebhetnew=np.delete(timebhet,x)
   Tavgbhetnew=np.delete(Tavgbhet,x)
   A0bhetnew=np.delete(A0bhet,x)
   logbhetnew=np.delete(logbhet,x)
   z=np.random.choice(np.arange(36),size=np.random.choice(9),replace=False)
   timenew=np.delete(time,z)
   Tavgnew=np.delete(Tavg,z)
   A0new=np.delete(A0,z)
   tpanew=np.delete(tpa,z)
try:
      popt,pcov=curve_fit(model1,np.array([timebhetnew,                                    Tavgbhetnew,
A0bhetnew]),logbhetnew,p0=guess)
      popt2,pcov2=curve_fit(model2,np.array([timenew, Tavgnew, A0new]),tpanew,p0=guess)
   except:
      err+=1

   myAsamples.append(popt[0])
   myAsamples2.append(popt2[0])
   myEAsamples.append(popt[1])
   myEAsamples2.append(popt2[1])
print(err)
```

```
plt.hist(myAsamples)
plt.figure(2)
plt.hist(myAsamples2)
plt.figure(3)
plt.hist(myEAsamples)
plt.figure(4)
plt.hist(myEAsamples2)
```
